# Civic and Political Engagement during the Multifaceted COVID‐19 Crisis

**DOI:** 10.1111/spsr.12446

**Published:** 2021-05-10

**Authors:** Endre Borbáth, Sophia Hunger, Swen Hutter, Ioana‐Elena Oana

**Affiliations:** ^1^ Freie Universität Berlin & WZB Berlin Social Science Center; ^2^ WZB Berlin Social Science Center; ^3^ European University Institute Florence

**Keywords:** COVID‐19, Civic Engagement, Political Engagement, Collective Action, Protest

## Abstract

Measures to cope with the COVID‐19 pandemic have put a sudden halt to street protests and other forms of citizen involvement in Europe. At the same time, the pandemic has increased the need for solidarity, motivating citizens to become involved on behalf of people at risk and the vulnerable more generally. This research note empirically examines the tension between the demobilisation and activation potential of the COVID‐19 crisis. Drawing on original survey data from seven Western European countries, we examine the extent, forms, and drivers of citizens’ engagement. Our findings show the remarkable persistence of pre‐existing political and civic engagement patterns. Concurrently, we show that threat perceptions triggered by the multifaceted COVID‐19 crisis have mobilized Europeans in the early phase of the pandemic. Similarly, the role of extreme ideological orientations in explaining (regular) political engagement indicates that the current situation may create its specific mobilisation potentials.

## Zusammenfassung

Die Massnahmen zur Bewältigung der COVID‐19 Pandemie haben Strassenprotesten und andere Formen des bürgerschaftlichen Engagements in Europa zunächst ein abruptes Ende gesetzt. Gleichzeitig hat die Pandemie den Bedarf an solidarischem Handeln erhöht und Bürger*innen dazu motiviert, sich für Risikogruppen und allgemein für Schwache einzusetzen. Diese empirische Studie untersucht das Spannungsverhältnis zwischen dem Demobilisierungs‐ und dem Aktivierungspotenzial der COVID‐19 Krise. Mittels Umfragedaten aus sieben westeuropäischen Ländern analysieren wir das Ausmass, die Formen und die Triebkräfte politischen und sozialen Engagements. Unsere Ergebnisse zeigen eine bemerkenswerte Beständigkeit bereits bestehender Muster. Gleichzeitig zeigen wir, dass durch die Vielschichtigkeit der Krise ausgelöste individuelle Bedrohungswahrnehmungen die Europäer*innen in der Frühphase der Pandemie mobilisiert haben. Ebenso besteht ein Zusammenhang zwischen radikalen ideologischen Orientierungen und (regelmässigem) politischem Engagement, der darauf hindeutet, dass die Pandemie ganz spezifische Mobilisierungspotenziale erzeugt.

## Résumé

Les mesures prises pour contrer la pandémie de COVID‐19 ont d'abord marqué un coup d’arrêt aux manifestations et aux autres formes de participation citoyenne en Europe. En même temps, la pandémie a renforcé le nécessité d'une solidarité plus forte, motivant les citoyens à s'engager pour les personnes à risque et les vulnérables en général. Cette note de recherche analyse la tension entre le potentiel de démobilisation et d'activation de la crise liée au COVID‐19. Sur la base de données de sondage pour sept pays d'Europe occidentale, nous examinons le degré, les formes et les motivations de la participation citoyenne. Nos résultats soulignent la persistance remarquable des modèles d'engagement politique et civique préexistants. En même temps, nous montrons que les perceptions de la menace provoquées par la crise COVID‐19 ont mobilisé les Européens dans la phase initiale de la pandémie. De même, les orientations idéologiques extrêmes expliquent l'engagement politique (régulier), ce qui indique que la situation de crise crée un potentiel de mobilisation spécifiques.

## Introduction

The ongoing COVID‐19 pandemic has placed European societies under unprecedented strain. The measures required to protect the health of the population involve high economic and personal sacrifice. Amid this dilemma, in spring 2020, most governments in Europe and beyond have adopted strict policies to curb the spread of the virus and alleviate pressure on their health systems. They also introduced immediate relief funds and various employment schemes to help the most economically affected sectors and individuals. Preliminary studies in political science explore how governments fare in solving this dilemma (e.g., Naumann et al. 2020; Petherick et al. 2020) or how citizens evaluate policy responses to this double health and economic threat posed by the pandemic (e.g., Bol et al. 2020; Esaiasson et al. 2020; Merkley 2020; Oana et al. 2021; Schraff 2020). This research note takes a more ‘bottom‐up’ perspective and shifts the focus from government action to civil society^1^ and citizens’ involvement (for related case studies, see Carlsen et al. 2020; Koos and Bertogg 2020; Zajak et al. 2020). Specifically, we aim to contribute to the scholarly literature on political behaviour and engagement during crises by asking whether threats and grievances triggered by the first wave of the pandemic had a (de‐)mobilizing effect on citizens in seven west European countries: France, Germany, Italy, the Netherlands, Spain, Sweden, and the United Kingdom.

While the COVID‐19 pandemic fits into a series of crises that European states underwent in the last decade, it also brings a new set of dilemmas to the fore. By creating a need to adopt efficient measures in order to contain infection rates quickly, the crisis confronted policymakers with questions such as: what is the level of risk societies can live with? To what extent do measures to protect public health justify a halt to the economy? Should public money cover private losses? What is the scope and limit of consensual decision making? Can the public be trusted to behave responsibly without the coercive power of the state? While at first a public health problem, the pandemic triggered a multifaceted crisis in many countries, beginning with the economic repercussions of curfews as well as (semi‐)lockdowns and impediments to large shares of national economies to (later on) governments’ legitimacy problems for unilaterally issuing newer and stricter measures (e.g., Herrera et al. 2020).

The multifaced character of the COVID‐19 crisis undoubtedly opens new research avenues, including its differing effects on individual‐level participation in politics and civil society. As a first step in this research agenda, we utilize a cross‐sectional survey conducted in seven West European countries to explore (a) the (de‐)mobilizing effects during the first phase of the COVID‐19 pandemic and (b) how threat perceptions and ideological predispositions have shaped Europeans’ engagement repertoire. By doing so, we can build on a rich body of scholarly work on how Europe’s recent crises – the financial and refugee crises in particular – have affected the dynamics of engagement (e.g., Grasso and Giugni 2016; Grasso and Lahusen 2020; Kern et al. 2015; Kurer et al. 2019). We argue that in response to the competing threats to health and economic status, both personal and societal, citizens across the ideological spectrum needed to re‐evaluate their civic and political engagement repertoire.

The distinction between civic and political engagement plays a crucial role in our analysis when it comes to mapping and exploring the push‐and‐pull dynamics associated with the multifaceted character of the COVID‐19 crisis. On the one hand, the newly introduced state measures have created the potential for political contestation, which, albeit for different reasons, have been challenged from both the left and right. At the same time, these measures have potentially shifted individual repertoires of engagement from forms that became less available (such as street protests or public gatherings) towards those easier to access (primarily in the online sphere) (Carlsen et al., 2020). On the other hand, the need for solidarity with and support for those at risk has increased. As in previous crises, civil society is called to provide social assistance and mobilize on behalf of those who are otherwise invisible or out of reach for policymakers (see, e.g., BMI 2020; Grasso and Lahusen 2020; Penner et al. 2005). From this perspective, civil society became crucial in maintaining social cohesion and providing channels for grassroots civic and political engagement.

This research note presents the first cut at these countervailing dynamics by focusing on the extent and drivers of individual engagement. Based on original survey data, we first describe how extensively and in what way Europeans involved themselves in the first few months of the COVID‐19 pandemic. Second, we use regression models to examine who became involved and who abstained. We are particularly interested in the role of health and economic threat perceptions (both personal and societal) and political ideology. These factors provide us with initial results about the unique and multifaceted character of the COVID‐19 crisis. We conclude the research note by raising questions for further research based on our preliminary empirical results.

## Measuring Engagement in the First Phase of the COVID‐19 Crisis

The data for this research note has been collected as part of a population survey conducted in seven Western European countries (France, Germany, Italy, the Netherlands, Spain, Sweden, and the UK) in the framework of the ERC Synergy Project SOLID (Policy Crisis and Crisis Politics: Sovereignty, Solidarity and Identity in the EU Post 2008).^2^ The questionnaire was administered following the first wave of COVID‐19, between June 5th and 22nd, 2020, on national samples using a quota sample design, with quotas for gender, age, area of residence, and education. The total sample size is 7'579; national sample sizes vary between 1'033 and 1'169

To measure civic and political engagement, we rely on a participation battery that measures whether respondents have taken part in seven types of activities^3^ on a four‐point scale (No; Yes, once; Yes, sometimes; Yes, often) in response to the COVID‐19 crisis. Given the question phrasing, we cover participation in the first phase of the crisis, from the beginning of the pandemic in Europe (associated with increasing infection numbers and the first lockdown measures in March 2020) until mid‐June.

Relying on exploratory factor analysis (see appendix A3), we group the items ‘help in the neighbourhood’ and ‘donating money’ into an indicator of civic engagement. The items ‘signing a petition,’ ‘contacting a politician,’ and ‘posting/sharing political content on the internet’ are combined into an indicator of political engagement. Given its more contentious nature, and the difficulties brought on by the pandemic, we separate ‘participating in a public demonstration’ into a third dependent variable.^4^ Our core measures capture whether respondents have participated in any of these forms (dichotomous). We also introduce a second measure to account for those who are *regularly* involved and answered that they had ‘often’ taken part in at least one of the forms (dichotomous) (for details, see appendix A1, A2).

## How Did Citizens Get Involved? Levels and Forms of Engagement across Europe

**Figure 1:**
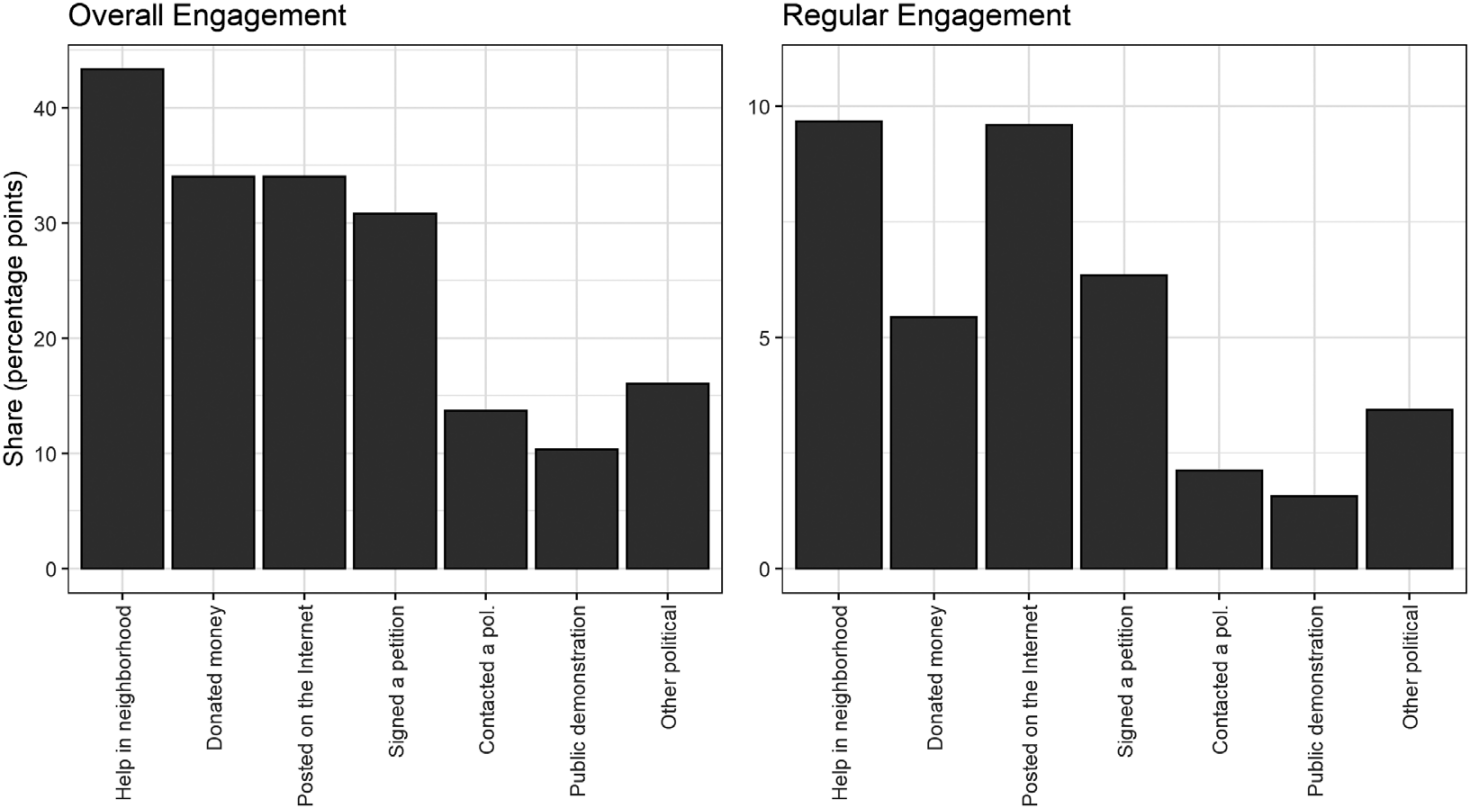
Levels of engagement for different action forms, country averages *Note:* See the levels by form and country in appendix A5, Table 1.

Figure 1 shows the average level of engagement for each item separately. The results underline that civic forms of engagement are more common than political forms of engagement. Helping out in the neighbourhood is the most widespread form of engagement in terms of occasional and regular participation (more than 43 percent of respondents did it at least once, and 9.7 percent did it regularly).^5^ However, other forms of engagement do not lag far behind: more than a third of the respondents report that they have donated money or posted something related to politics on the internet having to do with the COVID‐19 crisis (cf. Carlsen et al. 2020). Next to posting on the internet, signing a petition is the dominant form of political engagement. More than 30 percent of the sample indicates that they have at least once signed a petition. A little less than 15 percent answer that they have contacted a politician, and around 10 percent have participated in public demonstrations. While the shares of *regular* participants are far lower, the rank order of the different activities follows the same pattern. The exception is ‘donating money,’ which is less likely to evolve into a routine form of engagement. Moreover, the results are in sync with the latest wave of the European Social Survey (2018) for the seven countries included here and the activities specified in both surveys. In 2018, about 16 percent of the respondents in the seven countries contacted a politician, 30 percent signed a petition, and 9.8 percent demonstrated. In general, our results indicate sporadic but still fairly high levels of civic and political engagement during the first phase of the COVID‐19 crisis, manifesting levels and forms of behaviour previously prevalent across Western Europe.

**Figure 2:**
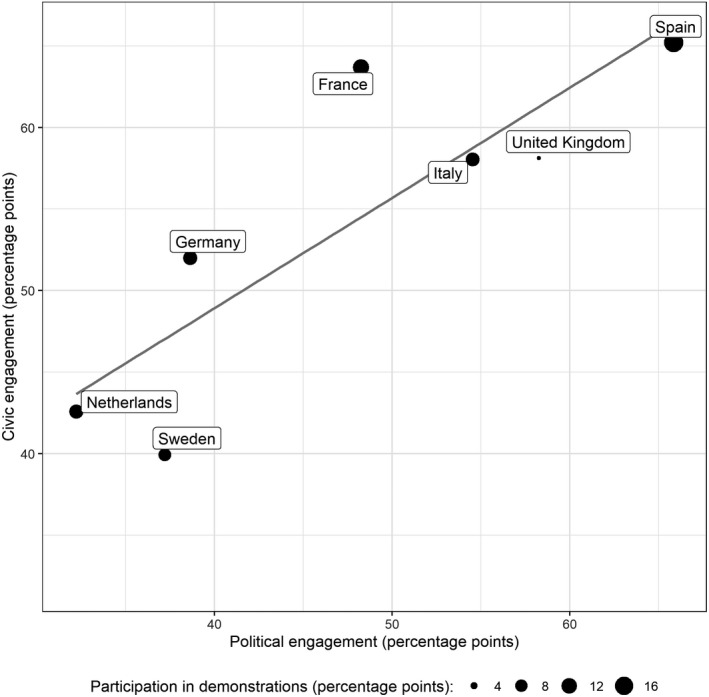
Scatterplot for political and civic engagement across countries *Note:* The Figure shows country averages. The size of the markers is proportional to the level of participation in demonstrations. For country‐specific values, see appendix A5, Figure 1.

Figure 2 presents country averages of overall political and civic engagement (see the previous section for the indicators). The size of the symbols is proportional to the level of participation in demonstrations. Spain is the country with the highest engagement level, followed by France, Italy, the United Kingdom, and Germany. Sweden and the Netherlands experience the lowest engagement rates. Regarding how people became involved, the survey results show that civic engagement has been slightly more common than political participation in most countries, potentially reflecting the immediate emergency aid in the health crisis. There is an even higher level of civic engagement in countries above the linear regression line than what a cross‐national comparison of political engagement leads us to expect. Most notably, in France, almost 60 percent of respondents reported having helped in the neighbourhood at least once during this period. In contrast, in countries below the line, political engagement is more dominant than civic forms with Sweden as the key example. The Swedish exceptionalism is potentially related to the lack of early and strict lockdown measures that might have led to less ‘need’ for civic forms of engagement otherwise required in the rest of Europe.

## Who Got Involved? Threat Perceptions and Ideology as Drivers of Engagement

In this section, we hone in on individual‐level variation. Exploring the multifaceted character of the COVID‐19 crisis, we emphasize the role of different types of perceived threats (health and economic) and on different levels (ego‐ and sociotropic) triggered by the pandemic. What makes the spread of COVID‐19 a fascinating context for exploring the dynamics of engagement is the unique mixture of threats that it brings about. The pandemic’s detrimental consequences and the measures to cope with health and economy‐related problems may generate reinforcing or competing sources of threat that citizens face. These threats might result in, on the one hand, a higher level of participation if citizens who feel threatened feel the need to ‘help out’ in terms of civic engagement and to make their ‘voices heard’ in terms of political engagement. On the other hand, the additional pressure of feeling threatened by the uncertain situation might result in a stronger inward focus and less engagement in civic and political forms. Given the exploratory nature of our analysis, we do not formulate expectations regarding the specific effects of these different types of threat on participation, but rather aim to empirically map associations visible in the first wave of the pandemic.

Compared to previous crises (such as the financial or refugee crisis), the multifaceted character of the COVID‐19 crisis also seems more ambiguous in terms of how it may relate to individual‐level ideological beliefs and affect well‐established links between ideology and participation. As the literature documents, left‐wing individuals are more likely to engage in non‐electoral forms of participation across Western Europe (e.g., Borbáth and Gessler 2020). At the same time, the uncertainty and contestation linked with the political measures to fight the crisis might have activated citizens from across the ideological spectrum. In the following, we will explore the effect of threat perceptions and ideology on the engagement repertoire of Europeans.

**Figure 3:**
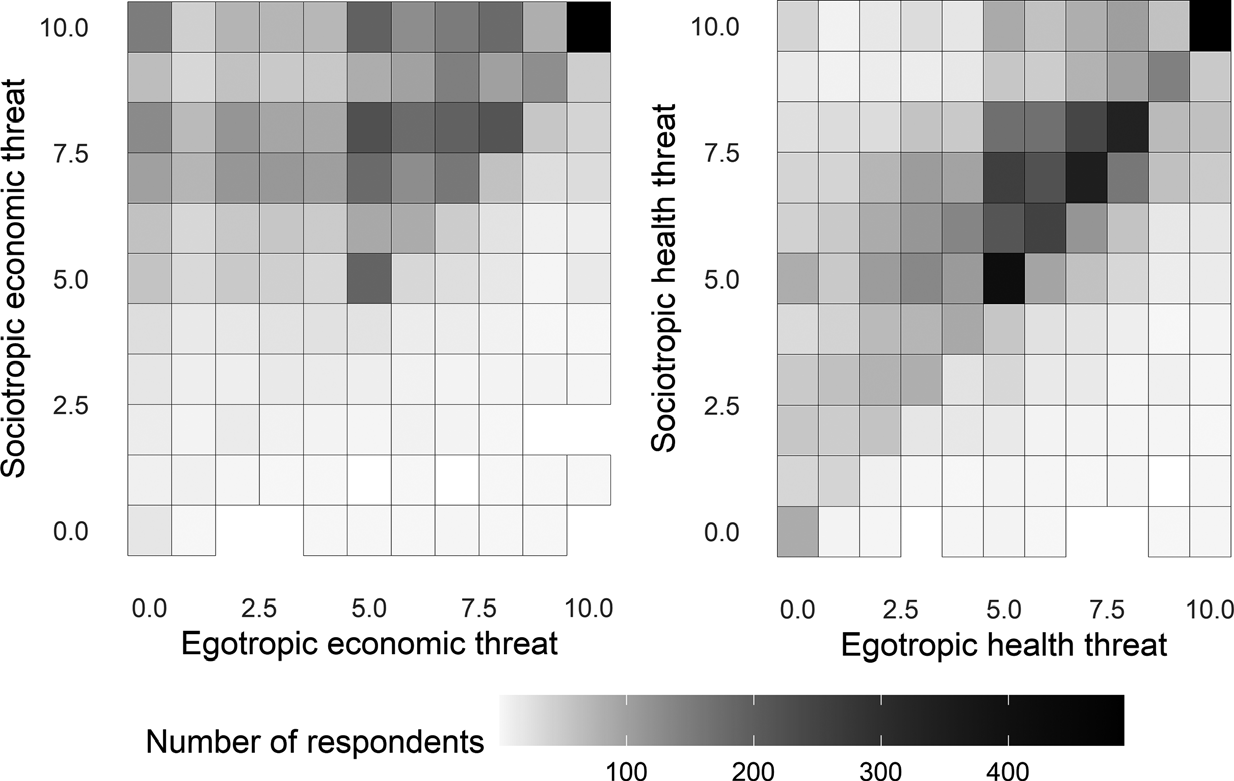
Heatmap for perceived economic and health threat on the societal and individual level *Note:* The color of the cells shows the number of respondents that rate the different threats on a 10‐point scale. For instance, 228 respondents choose to answer 5 when asked whether they perceive the pandemic as an economic threat to them both individually and for society.

Before presenting the regression analysis, Figure 3 maps the respondents’ threat perceptions in the seven countries during the first phase of the pandemic. The left‐hand panel shows threats regarding the economy, while the right‐hand side displays health threats. In each panel of the Figure, we distinguish between egotropic threats (i.e., threats that are perceived to be personal) shown on the x‐axis and sociotropic ones (i.e., perceived threats for society as a whole) displayed on the y‐axis. We observe quite some variance in the extent to which respondents consider COVID‐19 a threat to their own economic and health situation. In contrast, the overwhelming majority considers the economic and health repercussions as threatening to society as a whole (86.4 percent for the economy and 69.7 percent for health). Contrary to the disproportionate focus on COVID‐19 sceptics in the media, in most of these societies, the majority believes the spread of the virus is a threat to societal wellbeing in terms of economic and health risks (for the German case, see also Naumann et al. 2020).

In the next step, we model the individual drivers of engagement with logistic regressions. All coefficients (reported as odds ratios) are estimated based on separate models for civic engagement, political engagement, and participation in demonstrations. While we rely on country ‘fixed effects’ and clustered standard errors to isolate individual‐level differences, in appendix A4 we include country‐specific models that reflect the cross‐sectional differences in the findings we report, both for occasional and regular engagement.

We examine threat perceptions and ideology combined with other well‐established individual‐level determinants of political activity, such as socioeconomic characteristics and general political attitudes (e.g., Dalton et al. 2010; van Deth et al. 2007; Jenkins 1983; McAdam 1982; McCarthy and Zald 1977; Meyer 1993; Tarrow 1988). In line with the scholarly literature, we control for age, age squared, gender, education, having children, and the economic situation of the respondents, as well as for attitudinal characteristics, such as political interest, trust in the national government, and dissatisfaction with the national government’s handling of the pandemic. The complete regression tables with all estimates are included in the appendix A4 (for a simplified overview of significant independent variables by country, see Table 1 in appendix A4). Our results for these control variables go in the direction expected by the scholarly literature: younger, economically well‐off, more educated, and politically interested people are more likely to engage themselves politically or to take to the streets during the early months of the pandemic. For civic engagement, the results are similar, albeit age has no effect here. In addition, we find a strikingly consistent, positive association between the presence of children in one’s household and political and civic engagement. This indicates that while parents are seen as less involved during ‘normal times’ due to family responsibilities and related time constraints (McAdam 1986; Schussman and Soule 2005), they were more involved in the first period of the COVID‐19 pandemic, as part of the most affected groups by the lockdown measures.

While these findings are in line with previous literature, not all of them are consistent across countries. Increased activity for higher educated, politically interested, and economically well‐off respondents (Dalton et al. 2010; Rosenstone and Hansen 2003; Schlozman et al. 2012; Verba et al. 1995), as well as women’s lower likelihood to participate in demonstrations (Burns et al. 2001), are to be found in most countries under study. Regarding the effect of trust in the national government, we find a small (Braun and Hutter 2016; Hooghe and Marien 2013; Marien and Christensen 2013) but statistically significant positive effect on demonstrations, albeit only for some countries. The same applies to dissatisfaction with the government’s handling of the Coronavirus: our results indicate a positive association with political engagement in Germany, the UK, Sweden, and the Netherlands.

**Figure 4:**
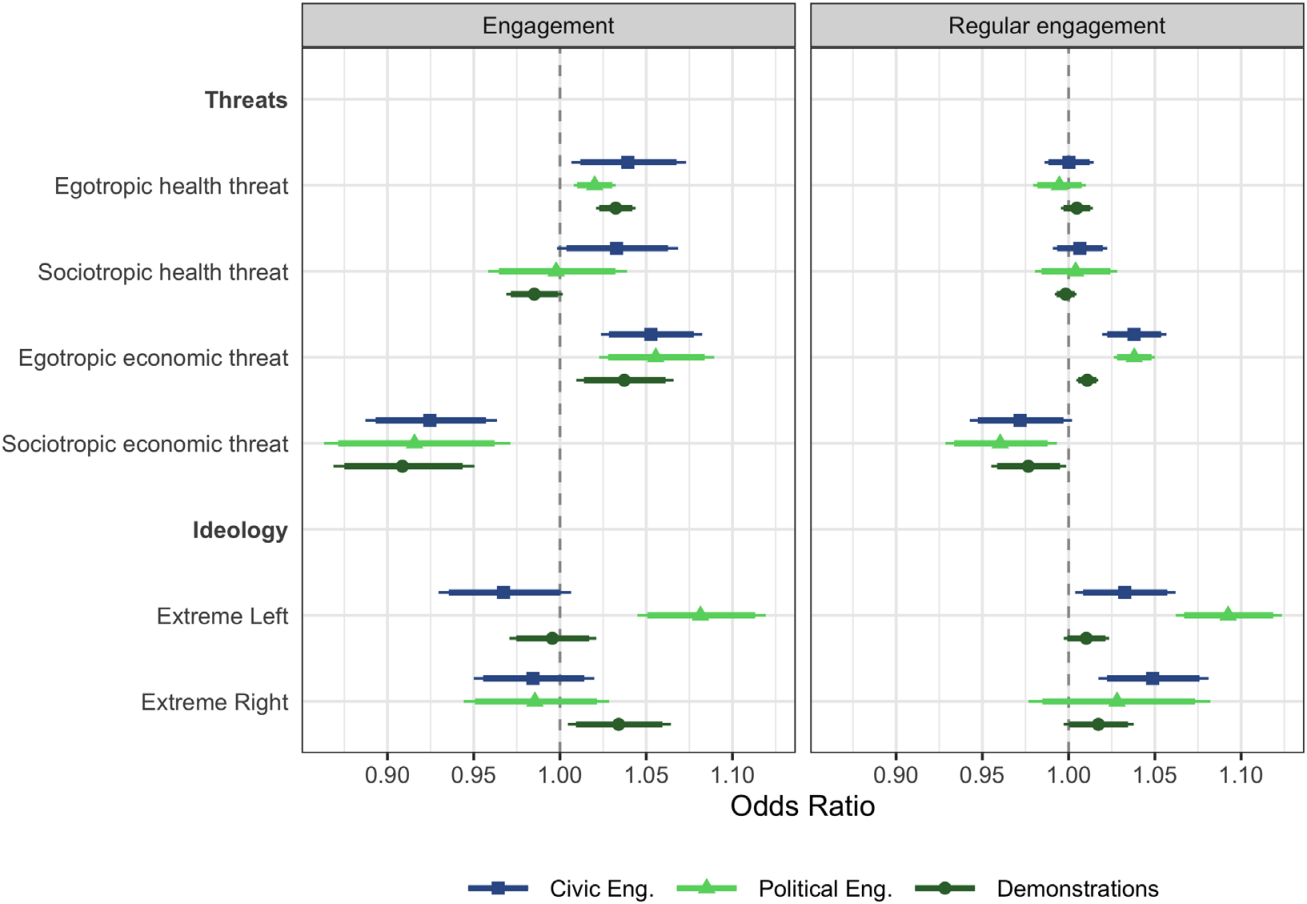
Coefficient plot of the effect of threats and ideology *Note:* The Figure shows odds ratios from three regression models, see the table in appendix A.4, Table 2. Outer confidence intervals are estimated at p<0.1 level, and inner confidence intervals are calculated at p<0.05 level. [Colour figure can be viewed at wileyonlinelibrary.com]

In the following, we focus on the effect of the four types of threats and ideology. To easily compare effect sizes, all four threat measures and the effect of left‐right self‐placement have been dichotomized.^6^ Figure 4 shows the coefficient plots for our respective regression models. We first focus on the effects on overall engagement, as shown by the panel on the left‐hand side.

As the Figure shows, *egotropic* threats tend to be more likely to mobilize citizens than sociotropic ones. We find that both health‐related and economy‐related egotropic threats are generally associated with a higher likelihood of becoming active in nearly all types of engagement. Regarding *sociotropic* threats, we find that more increased economic‐related threats are associated with a lower level of civic and political participation, whereas the effect of health threats is contingent on the form of participation. Sociotropic health threats seem to mobilize citizens in terms of civic engagement, demobilize in terms of less contentious political engagement, and have no statistically significant effect on participation in demonstrations. We believe the difference between the mobilizing effect of egotropic and the demobilizing effect of some of the sociotropic, particularly economic, threats is due to the timing of the survey. In June, the impact of the COVID‐19 crisis on national economies was not yet clear cut, and one could hope that the measures introduced to protect public health would phase out relatively quickly. Similarly, left‐right ideological beliefs are not statistically significantly related to civic engagement, whereas they colour political engagement. Extreme left respondents have been more likely to participate in less contentious forms of political engagement, whereas extreme right individuals have been more likely to participate in demonstrations during the first phase of the COVID‐19 crisis.

In terms of the cross‐national variation of these associations (see Table 1, A4), we find relative consistency regarding the mobilizing effect of *egotropic economic* threats and the demobilizing effect of *sociotropic economic* threats. However, the mobilizing effect of *egotropic health* threats is associated with the Netherlands and Sweden, two countries that first took a *laissez‐faire* approach to tackle the pandemic. The mobilizing effect of *sociotropic* health threats is the strongest in the UK and Sweden. The association between extreme left and less contentious forms of political engagement is strongest in the UK and Germany; in contrast, extreme right orientation is a driver of participation in demonstrations, especially in Sweden, the Netherlands, and Germany.

In terms of how these effects diverge when we model regular engagement (panel on the right‐hand side), we observe a weaker effect of threat perceptions associated with the crisis and stronger effects for ideology. Health threats – egotropic or sociotropic – do not significantly affect regular engagement, and economic threats have a weaker effect – mobilizing for egotropic and demobilizing for sociotropic – than for overall engagement. However, regular engagement, both civic and political, is associated with more extreme ideological views. Thus, longer‐term predispositions captured by ideological left‐right orientations seem to explain regular engagement better than short‐term crisis‐driven threat considerations.

## Conclusions

The research note has offered initial empirical evidence on the extent, forms, and drivers of citizens’ engagement in seven Western European countries during the first phase of the COVID‐19 crisis (i.e., March to mid‐June 2020). The results indicate that Europeans responded to the crisis by engaging themselves, although more sporadically than regularly. Reactions to COVID‐19 are similar to patterns documented by previous literature on the activation effect of crises and catastrophes: the pandemic mobilized existing solidarity practices in the form of both civic and political engagement (e.g., BMI 2020; Grasso and Giugni 2016; Grasso and Lahusen 2020; Kurer et al. 2019; Penner et al. 2005). Notably, large shares of the surveyed respondents indicated that they had helped in the neighbourhood (43 percent) and donated money (34 percent). Such forms of civic engagement were slightly more prevalent than political participation, although the COVID‐19 pandemic did not bring about a halt to the latter either (including public demonstrations). The smaller shares and the fact that the ‘usual suspects’ in terms of socioeconomic and attitudinal profiles became involved indicates that the pandemic tends to reinforce political inequalities rather than create new ones.

However, our in‐depth analysis of threat perceptions (health vs. economy and egotropic vs. sociotropic) and left‐right self‐placement as drivers of engagement uncovers that the current situation may create its specific mobilisation potentials. The results underscore the importance of distinguishing not only between the health and the economy as two sources of the threat the multifaceted COVID‐19 crisis triggers but also between their individual and societal dimensions. At least in the first phase of the crisis, egotropic threats appeared to be more influential than sociotropic ones in mobilizing participation. Similarly, economy‐related threats appeared to be more important than health threats. In addition, our results show that, unlike in the refugee crisis, civic engagement is not coloured by ideology, while political engagement is. However, the measures introduced to manage the pandemic result in a specific context in which the long‐established finding of left‐wing presence in demonstrations seems to hold no longer. During the early months of the pandemic, we observe a more substantial presence of the extreme right in demonstrations than of the political left.

Future research should consider in greater detail and with a prolonged study period how the multifaceted character of the COVID‐19 crisis changes such threat perceptions and grievances, which, in turn, might be activated by entrepreneurs in civil society and the party system. In general, our results on the individual level need to be connected to the systematic analysis of the organisational level. Given substantial restrictions on public assembly and a lack of digital infrastructures, it is an open empirical question to what extent the emerging forms of civic and political engagement have taken place outside of organized civil society. If a mismatch between individual demands for participation and organisational supply exists, it also remains to be seen how sustained and directed the immediate activation might be. Another mismatch worth investigating might also be found between the offered assistance and the emerging needs in society. So far, we have studied the individuals willing to help without distinguishing between participation ‘in the name of those most affected’ and participation ‘by those most affected’. It is equally important to analyse the extent to which civil society’s support matched actual needs and to identify those groups who might have felt left behind or overlooked. Our plea for better understanding how the *multifaceted* COVID‐19 crisis has given rise to specific mobilisation potentials should also be considered when political elites aim to channel and respond to the emerging mobilisation. Moreover, one should not misinterpret high civic and political engagement levels as yet another glorious moment for civil society. It is far from certain how organized civil society will (re‐)emerge in the post‐pandemic period and, thus, how sustained civic engagement may be without appropriate state measures that strengthen civil society in all its diversity.

### Open Research Badges

This article has earned Open Data and Open Materials badges for making publicly available the digitally‐shareable data necessary to reproduce the reported results. The data are available at https://forsbase.unil.ch.

## Supporting information

Supplementary Material

## Data Availability

The data that support the findings of this study are openly available in the Harvard Dataverse at “Replication Data for: Civic and Political Engagement during the Multifaceted COVID‐19 Crisis,” https://doi.org/10.7910/DVN/YNBJWK, Harvard Dataverse, V1, UNF:6:tYEIQBz8uWIfvpLy0Pdq6Q== [fileUNF].

## Footnotes

^1^
We adopt a sector‐specific logic and define civil society as all formal and informal voluntary associations that organize and channel citizens’ involvement in civic or social life and in more political forms of participation where issue advocacy takes a central stage (on the concept, see Edwards 2014).

^2^
The survey was conducted via CAWI methodology using the YouGov proprietary panel in all countries to recruit participants. See the appendix for the exact wording (A.1), descriptive statistics (A.2), and the sample sizes (appendix A5, Table 2).

^3^
Helped in the neighbourhood, donated money or material resources (e.g. food), signed a petition/collection of signatures, contacted a politician at federal, state or local level, posted or shared something about politics on the Internet, for example on blogs, by e‐mail, or in social media such as Facebook or Twitter, taken part in a public demonstration, become politically involved in a different way. See the exact wording in appendix A1.

^4^
We leave out the item “become politically involved in a different way” from the grouping given that it can refer to either civic or political engagement.

^5^
Koos and Bertogg (2020: 3) report very similar shares in their detailed study on Germany.

^6^
In the case of threat, all respondents who indicated a threat level of 6 or higher on the ten‐point scale are coded as 1, those who indicated a lower level of threat are coded as 0. In the case of ideology, all respondents who indicated on the 11‐point left‐right self‐placement scale a value of 0 or 1 are coded as extreme left, those who indicated a value of 9 or 10 are coded as extreme right.
