# Supplementary material for: Civic and Political Engagement during the Multifaceted COVID‐19 Crisis
Source: Schweiz Z Polit. 2021 May 10;27(2):311–24. doi: 10.1111/spsr.12446 (PMC8242449; doi:10.1111/spsr.12446)
Supplement: Supplementary file 1 — Supplementary Material [file SPSR-27-311-s001.docx]

Appendix:

Civic and Political Engagement during the Multifaceted COVID-19 Crisis

Endre Borbáth, Sophia Hunger, Swen Hutter, Ioana-Elena Oana

**Swiss Political Science Review**

Note: The data that support the findings of this study are openly available in the Harvard Dataverse at "Replication Data for: Civic and Political Engagement during the Multifaceted COVID-19 Crisis", <https://doi.org/10.7910/DVN/YNBJWK>, Harvard Dataverse, V1, UNF:6:tYEIQBz8uWIfvpLy0Pdq6Q== [fileUNF].

[A1: Wording of the main items used in the analysis 1](#_Toc62552371)

[A2: Descriptive statistics 3](#_Toc62552375)

[A3: Exploratory factor analysis of the participation items 5](#_Toc62552376)

[A4: Country specific analysis 8](#_Toc62552377)

[A5: Additional figures and tables 24](#_Toc62552378)

# A1: Wording of the main items used in the analysis

**Participation**

Due to the Coronavirus pandemic and its social, economic and political impact, have you undertaken any of the following activities?

1. No

2. Yes, once

3. Yes, sometimes

4. Yes, often

99 DK

(LIST) [RANDOMIZE ITEMS 1 TO 7]

1. helped in the neighborhood

2. donated money or material resources (e.g. food)

3. signed a petition/collection of signatures

4. contacted a politician at federal, state or local level

5. posted or shared something about politics on the Internet, for example on blogs, by e-mail, or in social media such as Facebook or Twitter

6. taken part in a public demonstration

7. become politically involved in a different way

**Threat perception**

How much of a threat, if any, is the Coronavirus outbreak for…:

| Not a threat at all |  |  |  |  |  |  |  |  |  | A major threat |
| --- | --- | --- | --- | --- | --- | --- | --- | --- | --- | --- |
| 0 | 1 | 2 | 3 | 4 | 5 | 6 | 7 | 8 | 9 | 10 |

(LIST) [RANDOMIZE ITEMS 1 TO 4]

1. The health of the [NATIONALITY] population as a whole
2. Your personal health
3. [COUNTRY]’s economy
4. Your household financial situation.

**Left-right self-placement**

In political matters, people sometimes talk of “left” and “right”. How would you place your views on this scale, where 0 means left and 10 means right?

| Left |  |  |  |  |  |  |  |  |  |  | Right |
| --- | --- | --- | --- | --- | --- | --- | --- | --- | --- | --- | --- |
| 0 |  | 1 | 2 | 3 | 4 | 5 | 6 | 7 | 8 | 9 | 10 |

98 I prefer not to say

99 DK

# A2: Descriptive statistics

Table 1: descriptive statistics for variables

| **Threat for individ. economic situation** |  |
| --- | --- |
| minimum | 0 |
| median (IQR) | 5.00 (3.00, 7.00) |
| mean (sd) | 5.10 (3.04) |
| maximum | 10 |
| **Threat for individ. health** |  |
| minimum | 0 |
| median (IQR) | 5.00 (3.00, 7.00) |
| mean (sd) | 5.36 (2.83) |
| maximum | 10 |
| **Societal health threat** |  |
| 0 | 1,981 (30) |
| 1 | 4,718 (70) |
| **Societal economic threat** |  |
| 0 | 792 (12) |
| 1 | 5,907 (88) |
| **Satisfaction with the gov. crisis handling** |  |
| minimum | 0 |
| median (IQR) | 6.00 (3.00, 8.00) |
| mean (sd) | 5.33 (3.05) |
| maximum | 10 |
| **Age** |  |
| minimum | 18 |
| median (IQR) | 50.00 (36.00, 63.00) |
| mean (sd) | 49.52 (16.34) |
| maximum | 95 |
| **Gender** |  |
| Male | 3,288 (49) |
| Female | 3,411 (51) |
| **Children** |  |
| minimum | 0 |
| median (IQR) | 0.00 (0.00, 1.00) |
| mean (sd) | 0.46 (0.89) |
| maximum | 6 |
| **Education** |  |
| lower than tertiary | 4,462 (67) |
| tertiary | 2,237 (33) |
| **Economic situation** |  |
| minimum | 1 |
| median (IQR) | 2.00 (1.00, 3.00) |
| mean (sd) | 2.10 (0.90) |
| maximum | 4 |
| **Extreme left** |  |
| minimum | 0 |
| median (IQR) | 0.00 (0.00, 0.00) |
| mean (sd) | 0.08 (0.27) |
| maximum | 1 |
| **Extreme right** |  |
| minimum | 0 |
| median (IQR) | 0.00 (0.00, 0.00) |
| mean (sd) | 0.08 (0.27) |
| maximum | 1 |
| **Political interest** |  |
| minimum | 1 |
| median (IQR) | 3.00 (2.00, 3.00) |
| mean (sd) | 2.76 (0.88) |
| maximum | 4 |
| **Trust in national government** |  |
| minimum | 0 |
| median (IQR) | 5.00 (2.00, 7.00) |
| mean (sd) | 4.78 (3.07) |
| maximum | 10 |

# A3: Exploratory factor analysis of the participation items

We used a Polychoric Correlation Matrix to calculate the factor structure and account for the participation measures not being continuous (4 point scales). The procedure yields a one factor solution:

Figure 1: Parallel Analysis Scree plots


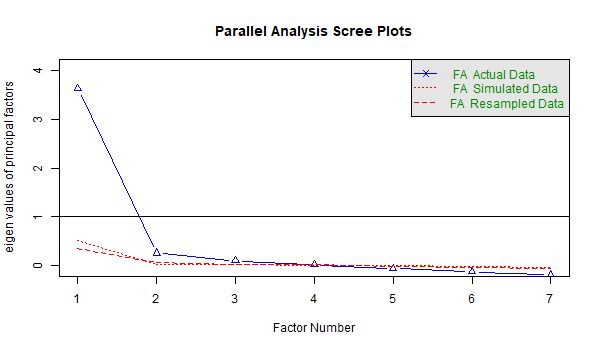


The factor solution it yields is composed from all engagement items and explains 0.52 proportion of the total variance with an eigenvalue of 3.64.

Figure 2: One factor solution


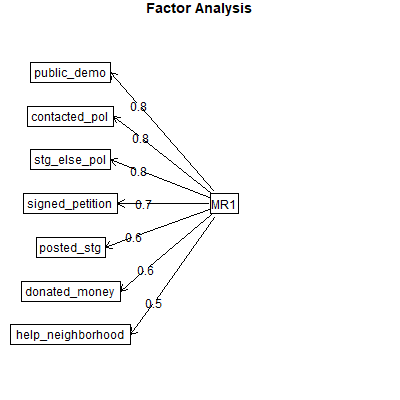


To examine the clustering between the different items we generate a two factors solution. In this case, the first factor explains 0.43, the second factor explains 0.16 proportion of the variance. The first factor has an eigenvalue of 2.98, the second factor has an eigenvalue of 1.1.

Figure 3: Two factor solution


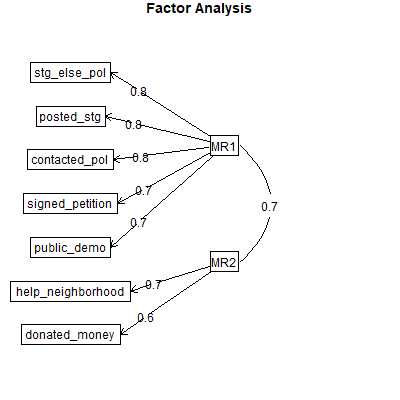


The solution generates a “political engagement” factor composed of the items attending public demonstrations, signing petitions, contacting a politician, posting something on the internet and being otherwise politically active, and a “social engagement” factor composed of the items helping in the neighborhood and donating money. From all items, donating money fits the least, but has a loading of 0.6, well above conventional thresholds.

The correlation coefficient between the two factors is 0.7. When we force the procedure to yield orthogonal factors (varimax rotation), the first factor explains 0.37 and the second factor explains 0.21 percent of the variance with an eigenvalue of 2.6 and respectively 1.48. In this solution all items load to the same extent (0.7).

Figure 4: Two factor solution with orthogonal factors (varimax rotation)


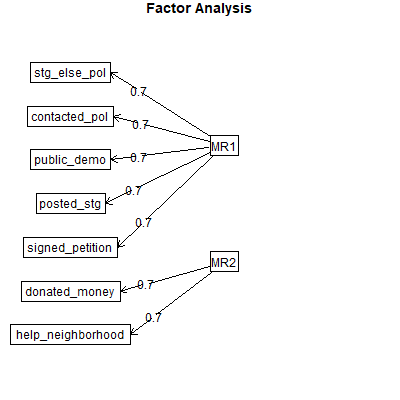


# A4: Country specific analysis

Table 1: Overview over direction and statistical significance of independent variables by country

| **Variable/DV** | **Civic** | **Political** | **Demon-stration** | **Civic - regular** | **Political - regular** | **Demo - regular** |
| --- | --- | --- | --- | --- | --- | --- |
| Ego. Health threat | SE*, NL* |  | SE*, NL* |  |  | FR* |
| Soc. Health threat | UK*, SE* |  |  |  |  |  |
| Ego. Eco. threat | SE*, NL*, FR* | SE*, NL*, DE*, FR* | SE*, NL*, FR* | NL* | SE*, NL* | UK*, NL* |
| Soc. Eco. Threat | DE, FR | DE, FR, ES | SE*, NL, DE, FR, ES | FR | FR | FR |
| Extreme left | UK | DE* |  |  | UK*, ES* |  |
| Extreme right |  |  | SE* | ES* | NL, ES* | NL*, DE* |
| age | UK*, NL, | NL, FR | SE, |  | UK, FR | SE |
| age^2 | NL* |  | SE* |  |  | SE* |
| gender | UK* | UK*, ES* | UK, NL, IT, DE | UK* |  |  |
| children | SE*, NL*, DE*, ES* | UK*, SE*, NL*, ES* | UK*, SE*, NL*, IT*, DE*, FR*, ES* | NL*, DE*, ES* | SE*, NL*, DE* | SE*, NL*, DE* |
| education | UK*, NL* |  | SE* |  | IT* |  |
| economic situation |  | SE*, IT* | IT* |  | UK* |  |
| political interest | UK*, SE*, FR*NL*, IT*, ES* | UK*, SE*, NL*, FR*IT*, DE*, ES* | NL*, IT*, FR*, ES* | UK*, SE*, NL*, FR*, | UK*, SE*, NL*, FR*IT*, DE*, ES* | NL*, IT*, FR*, ES* |
| trust in government | IT* | UK | SE*, NL* | IT* |  | SE*, IT* |
| government dissatisfaction |  | SE*, NL*, DE* | DE* | NL, | UK*, SE*, DE* |  |

Note: * indicates a positive direction, while no suffix signals a negative direction of the independent variable’s effect in a specific country

Table 2: Table for all countries

(models includes country fixed effects, which are not displayed here)

|  | **Demonstration** | **Political** | **Civic** | **Demonstration - regular** | **Political -regular** | **Civic -regular** |
| --- | --- | --- | --- | --- | --- | --- |
| intercept | 0.16^***^ | 0.17^*^ | 0.13 | 0.02^*^ | -0.08 | -0.06^**^ |
|  | (0.04) | (0.08) | (0.07) | (0.01) | (0.06) | (0.02) |
| age | -0.01^**^ | -0.01^**^ | 0.00 | -0.00^***^ | -0.00^**^ | 0.00 |
|  | (0.00) | (0.00) | (0.00) | (0.00) | (0.00) | (0.00) |
| age^2 | 0.00 | 0.00 | -0.00 | 0.00^**^ | 0.00^*^ | -0.00 |
|  | (0.00) | (0.00) | (0.00) | (0.00) | (0.00) | (0.00) |
| gender | -0.03^***^ | 0.04^*^ | 0.03^**^ | -0.00 | 0.02 | 0.03^*^ |
|  | (0.01) | (0.02) | (0.01) | (0.00) | (0.01) | (0.01) |
| children | 0.05^***^ | 0.04^***^ | 0.04^***^ | 0.01^***^ | 0.02^***^ | 0.02^**^ |
|  | (0.01) | (0.01) | (0.01) | (0.00) | (0.01) | (0.01) |
| education | 0.02^*^ | 0.01 | 0.06^***^ | 0.00 | 0.01^*^ | 0.02^***^ |
|  | (0.01) | (0.01) | (0.02) | (0.00) | (0.01) | (0.01) |
| economic situation | 0.02^***^ | 0.03^**^ | 0.02^**^ | 0.00 | 0.01^***^ | 0.00 |
|  | (0.00) | (0.01) | (0.01) | (0.00) | (0.00) | (0.01) |
| extreme left | 0.01 | 0.12^**^ | -0.00 | 0.01 | 0.11^***^ | 0.04^*^ |
|  | (0.01) | (0.04) | (0.03) | (0.01) | (0.02) | (0.02) |
| extreme right | 0.04^***^ | -0.02 | -0.02 | 0.02 | 0.03 | 0.04^**^ |
|  | (0.01) | (0.03) | (0.01) | (0.01) | (0.03) | (0.01) |
| political interest | 0.02^*^ | 0.14^***^ | 0.07^***^ | 0.01^***^ | 0.09^***^ | 0.03^***^ |
|  | (0.01) | (0.01) | (0.02) | (0.00) | (0.01) | (0.01) |
| trust in government | 0.01^***^ | -0.01 | 0.00 | 0.00 | -0.00 | 0.00 |
|  | (0.00) | (0.00) | (0.00) | (0.00) | (0.00) | (0.00) |
| government dissatisfaction | 0.00 | 0.02^*^ | 0.00 | -0.00 | 0.01^***^ | 0.00 |
|  | (0.00) | (0.01) | (0.00) | (0.00) | (0.00) | (0.00) |
| indiv. economic threat | 0.04^***^ | 0.08^***^ | 0.07^***^ | 0.01^***^ | 0.05^***^ | 0.04^***^ |
|  | (0.01) | (0.02) | (0.02) | (0.00) | (0.01) | (0.01) |
| indiv. health threat | 0.03^***^ | 0.02^***^ | 0.04^*^ | 0.01 | -0.00 | 0.00 |
|  | (0.01) | (0.00) | (0.02) | (0.00) | (0.01) | (0.01) |
| soc. economic threat | -0.10^***^ | -0.06^*^ | -0.06^*^ | -0.02^*^ | -0.03^*^ | -0.02 |
|  | (0.02) | (0.03) | (0.03) | (0.01) | (0.02) | (0.02) |
| soc. health threat | -0.01^**^ | 0.03 | 0.05^**^ | -0.00 | 0.01 | 0.01 |
|  | (0.00) | (0.02) | (0.01) | (0.00) | (0.01) | (0.01) |
| AIC | 2253.87 | 9034.20 | 9438.56 | -9296.18 | 4451.55 | 4363.06 |
| BIC | 2369.64 | 9149.97 | 9554.33 | -9180.41 | 4567.32 | 4478.83 |
| Log Likelihood | -1109.93 | -4500.10 | -4702.28 | 4665.09 | -2208.78 | -2164.53 |
| Deviance | 541.79 | 1490.73 | 1583.49 | 96.62 | 752.16 | 742.28 |
| Num. obs. | 6699 | 6699 | 6699 | 6699 | 6699 | 6699 |
| ^***^p < 0.001; ^**^p < 0.01; ^*^p < 0.05 | | | | | | |

Figure 1: Odds ratios of main variables for the UK


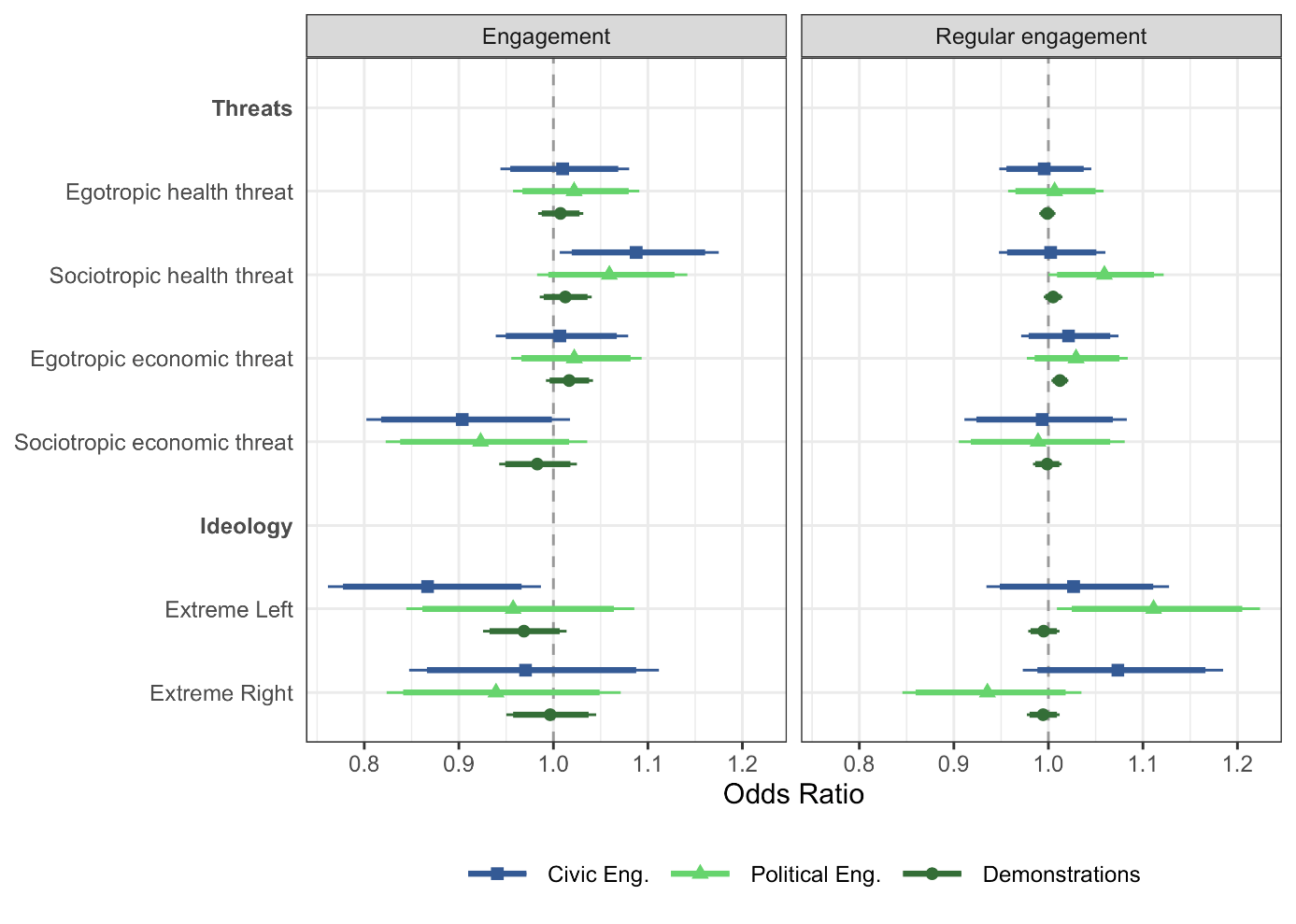


Table 3 models for the UK

|  | **Demonstration** | **Political** | **Civic** | **Demonstration - regular** | **Political -regular** | **Civic -regular** |
| --- | --- | --- | --- | --- | --- | --- |
| intercept | 0.07 | 0.25 | -0.11 | 0.01 | -0.04 | -0.12 |
|  | (0.06) | (0.16) | (0.17) | (0.02) | (0.12) | (0.12) |
| age | -0.00 | -0.00 | 0.01^*^ | -0.00 | -0.01^*^ | 0.00 |
|  | (0.00) | (0.01) | (0.01) | (0.00) | (0.00) | (0.00) |
| age^2 | 0.00 | -0.00 | -0.00 | 0.00 | 0.00 | -0.00 |
|  | (0.00) | (0.00) | (0.00) | (0.00) | (0.00) | (0.00) |
| gender | -0.03^**^ | 0.12^***^ | 0.09^**^ | -0.01 | 0.02 | 0.05^*^ |
|  | (0.01) | (0.03) | (0.03) | (0.00) | (0.02) | (0.02) |
| children | 0.01 | 0.04^*^ | 0.05^**^ | 0.00 | -0.01 | 0.00 |
|  | (0.01) | (0.02) | (0.02) | (0.00) | (0.01) | (0.01) |
| education | -0.00 | -0.02 | 0.11^***^ | 0.00 | 0.00 | 0.01 |
|  | (0.01) | (0.03) | (0.03) | (0.00) | (0.02) | (0.02) |
| economic situation | 0.01 | 0.00 | 0.01 | -0.00 | 0.03^*^ | 0.01 |
|  | (0.01) | (0.02) | (0.02) | (0.00) | (0.02) | (0.02) |
| extreme left | -0.03 | -0.04 | -0.14^*^ | -0.00 | 0.11^*^ | 0.03 |
|  | (0.02) | (0.06) | (0.07) | (0.01) | (0.05) | (0.05) |
| extreme right | -0.00 | -0.06 | -0.03 | -0.01 | -0.07 | 0.07 |
|  | (0.02) | (0.07) | (0.07) | (0.01) | (0.05) | (0.05) |
| political interest | 0.00 | 0.16^***^ | 0.12^***^ | 0.00 | 0.11^***^ | 0.04^**^ |
|  | (0.01) | (0.02) | (0.02) | (0.00) | (0.01) | (0.01) |
| trust in government | 0.00 | -0.02^*^ | 0.00 | -0.00 | -0.01 | 0.00 |
|  | (0.00) | (0.01) | (0.01) | (0.00) | (0.01) | (0.01) |
| government dissatisfaction | 0.00 | 0.00 | -0.00 | -0.00 | 0.01^*^ | 0.00 |
|  | (0.00) | (0.01) | (0.01) | (0.00) | (0.01) | (0.01) |
| indiv. economic threat | 0.02 | 0.02 | 0.01 | 0.01^**^ | 0.03 | 0.02 |
|  | (0.01) | (0.03) | (0.04) | (0.00) | (0.03) | (0.03) |
| indiv. health threat | 0.01 | 0.02 | 0.01 | -0.00 | 0.01 | -0.00 |
|  | (0.01) | (0.03) | (0.03) | (0.00) | (0.03) | (0.02) |
| soc. economic threat | -0.02 | -0.08 | -0.10 | -0.00 | -0.01 | -0.01 |
|  | (0.02) | (0.06) | (0.06) | (0.01) | (0.05) | (0.04) |
| soc. health threat | 0.01 | 0.06 | 0.08^*^ | 0.00 | 0.06 | 0.00 |
|  | (0.01) | (0.04) | (0.04) | (0.01) | (0.03) | (0.03) |
| AIC | -718.76 | 1333.27 | 1397.40 | -2764.20 | 800.10 | 748.26 |
| BIC | -635.13 | 1416.90 | 1481.03 | -2680.56 | 883.73 | 831.89 |
| Log Likelihood | 376.38 | -649.63 | -681.70 | 1399.10 | -383.05 | -357.13 |
| Deviance | 27.49 | 208.86 | 222.52 | 3.64 | 123.32 | 117.16 |
| Num. obs. | 1012 | 1012 | 1012 | 1012 | 1012 | 1012 |
| ^***^p < 0.001; ^**^p < 0.01; ^*^p < 0.05 | | | | | | |

Figure 2: Odds ratios of main variables for Sweden


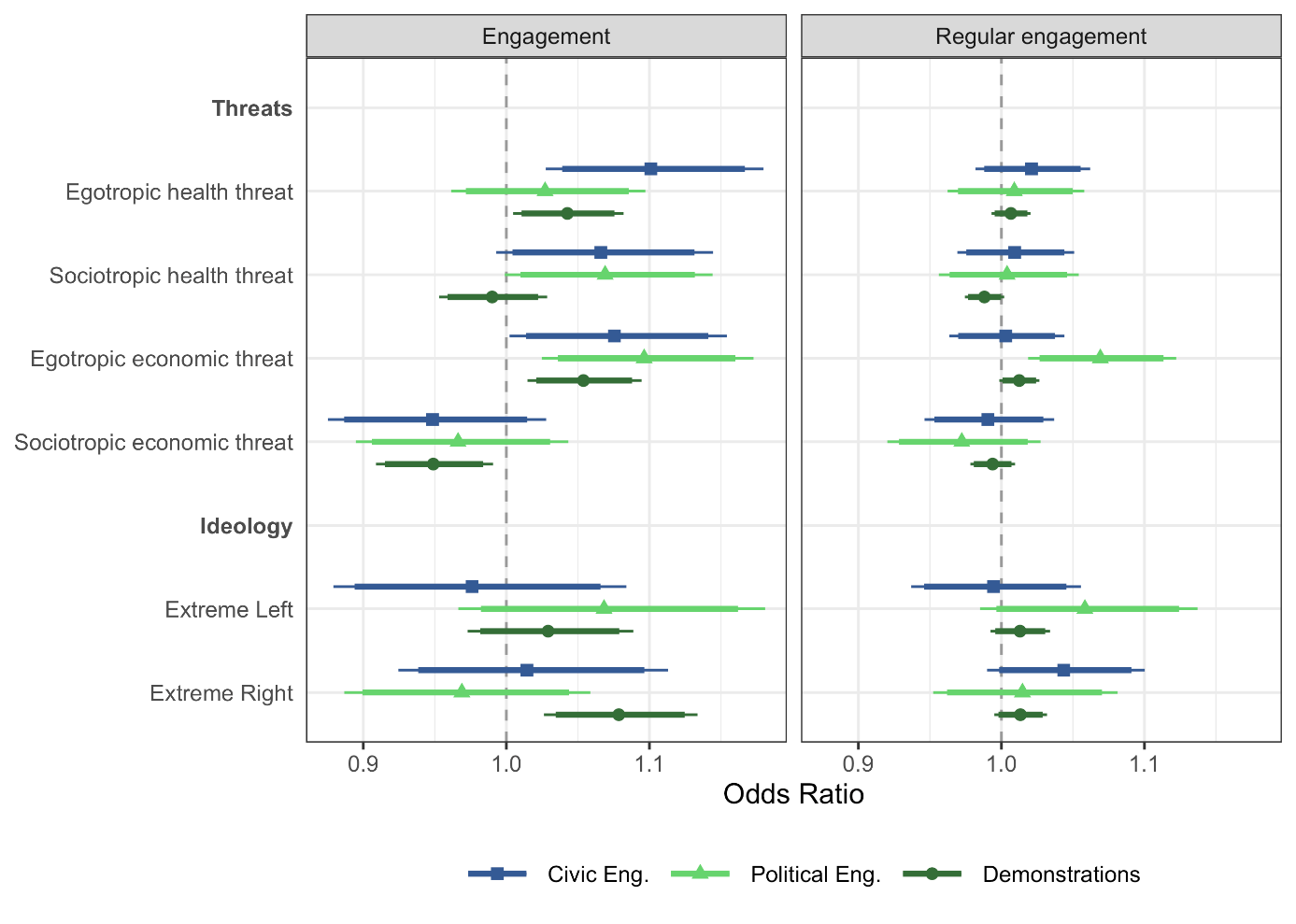


Table 4 models for Sweden

|  | **Demonstration** | **Political** | **Civic** | **Demonstration - regular** | **Political -regular** | **Civic -regular** |
| --- | --- | --- | --- | --- | --- | --- |
| intercept | 0.32^***^ | -0.27 | -0.08 | 0.04 | -0.32^**^ | -0.01 |
|  | (0.09) | (0.16) | (0.17) | (0.03) | (0.12) | (0.10) |
| age | -0.02^***^ | -0.00 | 0.01 | -0.00^**^ | 0.00 | 0.00 |
|  | (0.00) | (0.01) | (0.01) | (0.00) | (0.00) | (0.00) |
| age^2 | 0.00^***^ | 0.00 | -0.00 | 0.00^**^ | -0.00 | -0.00 |
|  | (0.00) | (0.00) | (0.00) | (0.00) | (0.00) | (0.00) |
| gender | -0.02 | 0.01 | 0.04 | 0.00 | -0.02 | 0.02 |
|  | (0.02) | (0.03) | (0.03) | (0.01) | (0.02) | (0.02) |
| children | 0.04^***^ | 0.06^***^ | 0.09^***^ | 0.02^***^ | 0.03^**^ | 0.01 |
|  | (0.01) | (0.02) | (0.02) | (0.00) | (0.01) | (0.01) |
| education | 0.03^*^ | 0.01 | 0.06 | -0.00 | -0.01 | 0.01 |
|  | (0.02) | (0.03) | (0.03) | (0.01) | (0.02) | (0.02) |
| economic situation | 0.02 | 0.06^***^ | 0.01 | -0.00 | 0.02 | 0.01 |
|  | (0.01) | (0.02) | (0.02) | (0.00) | (0.01) | (0.01) |
| extreme left | 0.03 | 0.07 | -0.02 | 0.01 | 0.06 | -0.01 |
|  | (0.03) | (0.05) | (0.05) | (0.01) | (0.04) | (0.03) |
| extreme right | 0.08^**^ | -0.03 | 0.01 | 0.01 | 0.01 | 0.04 |
|  | (0.03) | (0.05) | (0.05) | (0.01) | (0.03) | (0.03) |
| political interest | 0.01 | 0.16^***^ | 0.07^***^ | 0.00 | 0.10^***^ | 0.03^*^ |
|  | (0.01) | (0.02) | (0.02) | (0.00) | (0.01) | (0.01) |
| trust in government | 0.01^***^ | 0.01 | 0.01 | 0.00^*^ | 0.01 | -0.00 |
|  | (0.00) | (0.01) | (0.01) | (0.00) | (0.01) | (0.00) |
| government dissatisfaction | 0.00 | 0.02^*^ | -0.00 | 0.00 | 0.02^**^ | -0.00 |
|  | (0.00) | (0.01) | (0.01) | (0.00) | (0.01) | (0.00) |
| indiv. economic threat | 0.05^**^ | 0.09^**^ | 0.07^*^ | 0.01 | 0.07^**^ | 0.00 |
|  | (0.02) | (0.03) | (0.04) | (0.01) | (0.02) | (0.02) |
| indiv. health threat | 0.04^*^ | 0.03 | 0.10^**^ | 0.01 | 0.01 | 0.02 |
|  | (0.02) | (0.03) | (0.04) | (0.01) | (0.02) | (0.02) |
| soc. economic threat | -0.05^*^ | -0.03 | -0.05 | -0.01 | -0.03 | -0.01 |
|  | (0.02) | (0.04) | (0.04) | (0.01) | (0.03) | (0.02) |
| soc. health threat | -0.01 | 0.07 | 0.06 | -0.01 | 0.00 | 0.01 |
|  | (0.02) | (0.03) | (0.04) | (0.01) | (0.02) | (0.02) |
| AIC | 113.12 | 1259.79 | 1349.04 | -1883.60 | 598.96 | 230.53 |
| BIC | 196.42 | 1343.09 | 1432.33 | -1800.31 | 682.26 | 313.82 |
| Log Likelihood | -39.56 | -612.90 | -657.52 | 958.80 | -282.48 | -98.26 |
| Deviance | 62.80 | 199.53 | 218.31 | 8.39 | 102.49 | 70.70 |
| Num. obs. | 992 | 992 | 992 | 992 | 992 | 992 |
| ^***^p < 0.001; ^**^p < 0.01; ^*^p < 0.05 | | | | | | |

Figure 3: Odds ratios of main variables for the Netherlands


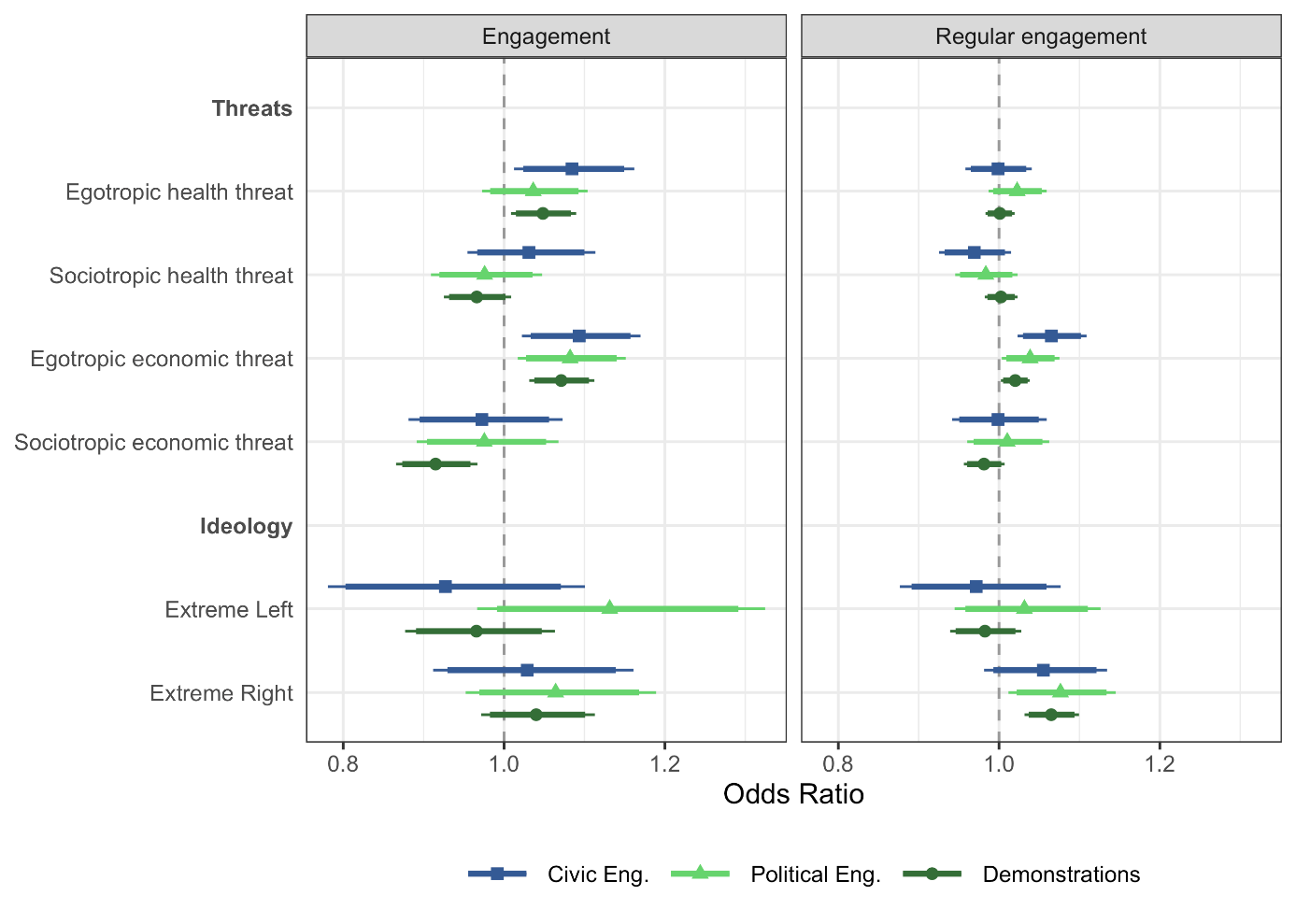


Table 5 models for the Netherlands

|  | **Demonstration** | **Political** | **Civic** | **Demonstration - regular** | **Political -regular** | **Civic -regular** |
| --- | --- | --- | --- | --- | --- | --- |
| intercept | 0.13 | 0.28 | 0.38^*^ | 0.03 | -0.13 | 0.03 |
|  | (0.09) | (0.15) | (0.16) | (0.04) | (0.08) | (0.10) |
| age | -0.00 | -0.01^**^ | -0.01^*^ | -0.00 | -0.00 | -0.00 |
|  | (0.00) | (0.00) | (0.01) | (0.00) | (0.00) | (0.00) |
| age^2 | 0.00 | 0.00 | 0.00^*^ | 0.00 | 0.00 | 0.00 |
|  | (0.00) | (0.00) | (0.00) | (0.00) | (0.00) | (0.00) |
| gender | -0.06^**^ | -0.04 | -0.01 | -0.00 | 0.01 | -0.02 |
|  | (0.02) | (0.03) | (0.03) | (0.01) | (0.02) | (0.02) |
| children | 0.06^***^ | 0.04^*^ | 0.05^**^ | 0.02^***^ | 0.03^**^ | 0.04^***^ |
|  | (0.01) | (0.02) | (0.02) | (0.00) | (0.01) | (0.01) |
| education | 0.02 | -0.01 | 0.09^**^ | 0.01 | 0.01 | 0.03 |
|  | (0.02) | (0.03) | (0.03) | (0.01) | (0.02) | (0.02) |
| economic situation | 0.01 | 0.03 | -0.02 | -0.00 | 0.02 | 0.00 |
|  | (0.01) | (0.02) | (0.02) | (0.01) | (0.01) | (0.01) |
| extreme left | -0.03 | 0.12 | -0.08 | -0.02 | 0.03 | -0.03 |
|  | (0.05) | (0.08) | (0.09) | (0.02) | (0.04) | (0.05) |
| extreme right | 0.04 | 0.06 | 0.03 | 0.06^***^ | 0.07^*^ | 0.05 |
|  | (0.03) | (0.06) | (0.06) | (0.02) | (0.03) | (0.04) |
| political interest | 0.04^**^ | 0.14^***^ | 0.10^***^ | 0.01^**^ | 0.07^***^ | 0.06^***^ |
|  | (0.01) | (0.02) | (0.02) | (0.01) | (0.01) | (0.01) |
| trust in government | 0.01^*^ | 0.00 | 0.01 | 0.00 | -0.00 | 0.00 |
|  | (0.01) | (0.01) | (0.01) | (0.00) | (0.00) | (0.01) |
| government dissatisfaction | 0.00 | 0.02^*^ | -0.01 | -0.00 | 0.00 | -0.01^*^ |
|  | (0.01) | (0.01) | (0.01) | (0.00) | (0.01) | (0.01) |
| indiv. economic threat | 0.07^***^ | 0.08^*^ | 0.09^**^ | 0.02^*^ | 0.04^*^ | 0.06^**^ |
|  | (0.02) | (0.03) | (0.03) | (0.01) | (0.02) | (0.02) |
| indiv. health threat | 0.05^*^ | 0.04 | 0.08^*^ | 0.00 | 0.02 | -0.00 |
|  | (0.02) | (0.03) | (0.04) | (0.01) | (0.02) | (0.02) |
| soc. economic threat | -0.09^**^ | -0.02 | -0.03 | -0.02 | 0.01 | -0.00 |
|  | (0.03) | (0.05) | (0.05) | (0.01) | (0.03) | (0.03) |
| soc. health threat | -0.03 | -0.02 | 0.03 | 0.00 | -0.02 | -0.03 |
|  | (0.02) | (0.04) | (0.04) | (0.01) | (0.02) | (0.02) |
| AIC | 207.32 | 1209.25 | 1382.88 | -1337.31 | 20.42 | 339.77 |
| BIC | 291.04 | 1292.97 | 1466.59 | -1253.59 | 104.14 | 423.49 |
| Log Likelihood | -86.66 | -587.63 | -674.44 | 685.65 | 6.79 | -152.88 |
| Deviance | 70.27 | 188.21 | 223.25 | 15.39 | 58.48 | 80.05 |
| Num. obs. | 1017 | 1017 | 1017 | 1017 | 1017 | 1017 |
| ^***^p < 0.001; ^**^p < 0.01; ^*^p < 0.05 | | | | | | |

Figure 4: Odds ratios of main variables for Italy


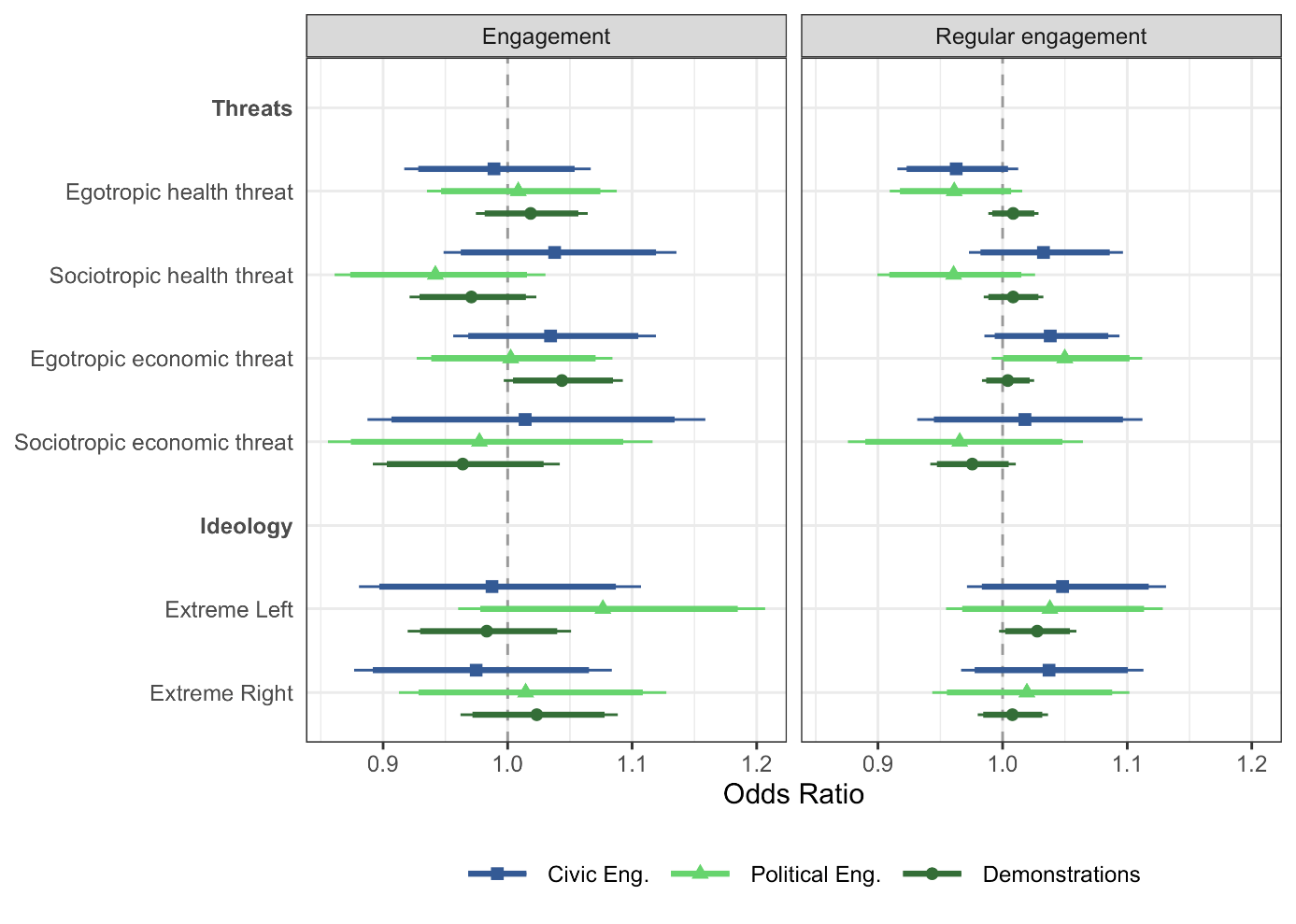


Table 6 models for Italy

|  | **Demonstration** | **Political** | **Civic** | **Demonstration - regular** | **Political -regular** | **Civic -regular** |
| --- | --- | --- | --- | --- | --- | --- |
| intercept | 0.02 | 0.34 | 0.25 | -0.03 | 0.15 | 0.04 |
|  | (0.10) | (0.18) | (0.18) | (0.05) | (0.13) | (0.12) |
| age | -0.00 | -0.01 | -0.00 | -0.00 | -0.01 | -0.01 |
|  | (0.00) | (0.01) | (0.01) | (0.00) | (0.00) | (0.00) |
| age^2 | 0.00 | 0.00 | -0.00 | 0.00 | 0.00 | 0.00 |
|  | (0.00) | (0.00) | (0.00) | (0.00) | (0.00) | (0.00) |
| gender | -0.04^*^ | 0.01 | 0.01 | -0.01 | 0.01 | 0.02 |
|  | (0.02) | (0.03) | (0.03) | (0.01) | (0.02) | (0.02) |
| children | 0.05^***^ | 0.02 | 0.02 | 0.01 | 0.02 | 0.01 |
|  | (0.01) | (0.02) | (0.02) | (0.01) | (0.01) | (0.01) |
| education | -0.01 | 0.07 | 0.01 | 0.01 | 0.09^**^ | 0.02 |
|  | (0.03) | (0.05) | (0.05) | (0.01) | (0.03) | (0.03) |
| economic situation | 0.02^*^ | 0.04^*^ | 0.01 | 0.00 | 0.01 | 0.01 |
|  | (0.01) | (0.02) | (0.02) | (0.00) | (0.01) | (0.01) |
| extreme left | -0.02 | 0.07 | -0.01 | 0.03 | 0.04 | 0.05 |
|  | (0.03) | (0.06) | (0.06) | (0.02) | (0.04) | (0.04) |
| extreme right | 0.02 | 0.01 | -0.03 | 0.01 | 0.02 | 0.04 |
|  | (0.03) | (0.05) | (0.05) | (0.01) | (0.04) | (0.04) |
| political interest | 0.03^**^ | 0.14^***^ | 0.09^***^ | 0.01^*^ | 0.07^***^ | 0.02 |
|  | (0.01) | (0.02) | (0.02) | (0.01) | (0.01) | (0.01) |
| trust in government | 0.01 | 0.01 | 0.02^**^ | 0.00^*^ | 0.00 | 0.01^*^ |
|  | (0.00) | (0.01) | (0.01) | (0.00) | (0.01) | (0.01) |
| government dissatisfaction | 0.00 | 0.01 | 0.00 | 0.00 | 0.00 | 0.01 |
|  | (0.00) | (0.01) | (0.01) | (0.00) | (0.01) | (0.01) |
| indiv. economic threat | 0.04 | 0.00 | 0.03 | 0.00 | 0.05 | 0.04 |
|  | (0.02) | (0.04) | (0.04) | (0.01) | (0.03) | (0.03) |
| indiv. health threat | 0.02 | 0.01 | -0.01 | 0.01 | -0.04 | -0.04 |
|  | (0.02) | (0.04) | (0.04) | (0.01) | (0.03) | (0.03) |
| soc. economic threat | -0.04 | -0.02 | 0.01 | -0.02 | -0.04 | 0.02 |
|  | (0.04) | (0.07) | (0.07) | (0.02) | (0.05) | (0.05) |
| soc. health threat | -0.03 | -0.06 | 0.04 | 0.01 | -0.04 | 0.03 |
|  | (0.03) | (0.05) | (0.05) | (0.01) | (0.03) | (0.03) |
| AIC | 273.93 | 1261.87 | 1266.20 | -1179.35 | 692.68 | 518.04 |
| BIC | 355.93 | 1343.87 | 1348.20 | -1097.36 | 774.67 | 600.04 |
| Log Likelihood | -119.97 | -613.94 | -616.10 | 606.68 | -329.34 | -242.02 |
| Deviance | 69.31 | 203.08 | 204.04 | 14.26 | 109.31 | 90.40 |
| Num. obs. | 919 | 919 | 919 | 919 | 919 | 919 |
| ^***^p < 0.001; ^**^p < 0.01; ^*^p < 0.05 | | | | | | |

Figure 5: Odds ratios of main variables for Germany


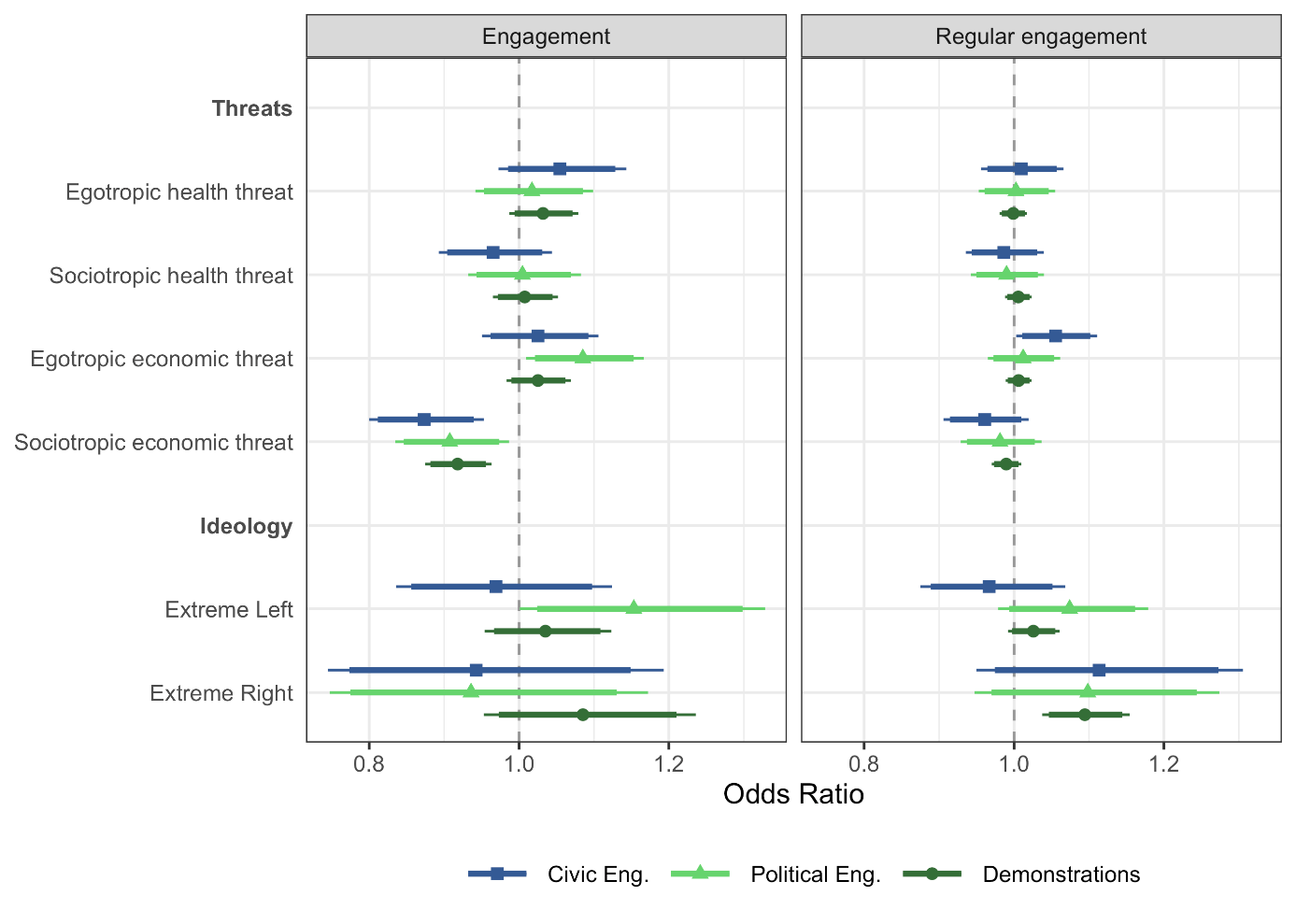


Table 7 models for Germany

|  | **Demonstration** | **Political** | **Civic** | **Demonstration - regular** | **Political -regular** | **Civic -regular** |
| --- | --- | --- | --- | --- | --- | --- |
| intercept | 0.21^*^ | 0.11 | 0.19 | 0.02 | 0.00 | -0.13 |
|  | (0.10) | (0.17) | (0.18) | (0.04) | (0.11) | (0.12) |
| age | -0.01 | -0.01 | 0.01 | -0.00 | -0.01 | 0.01 |
|  | (0.00) | (0.01) | (0.01) | (0.00) | (0.00) | (0.00) |
| age^2 | 0.00 | 0.00 | -0.00 | 0.00 | 0.00 | -0.00 |
|  | (0.00) | (0.00) | (0.00) | (0.00) | (0.00) | (0.00) |
| gender | -0.05^**^ | 0.05 | 0.01 | 0.00 | -0.00 | -0.01 |
|  | (0.02) | (0.03) | (0.03) | (0.01) | (0.02) | (0.02) |
| children | 0.04^**^ | 0.03 | 0.05^*^ | 0.01^*^ | 0.03^*^ | 0.05^***^ |
|  | (0.01) | (0.02) | (0.02) | (0.01) | (0.01) | (0.02) |
| education | -0.00 | -0.02 | 0.03 | 0.00 | -0.01 | 0.01 |
|  | (0.02) | (0.04) | (0.04) | (0.01) | (0.02) | (0.03) |
| economic situation | 0.02 | 0.03 | -0.00 | 0.00 | 0.02 | -0.02 |
|  | (0.01) | (0.02) | (0.02) | (0.00) | (0.01) | (0.01) |
| extreme left | 0.03 | 0.14^*^ | -0.03 | 0.03 | 0.07 | -0.03 |
|  | (0.04) | (0.07) | (0.08) | (0.02) | (0.05) | (0.05) |
| extreme right | 0.08 | -0.07 | -0.06 | 0.09^***^ | 0.09 | 0.11 |
|  | (0.07) | (0.11) | (0.12) | (0.03) | (0.08) | (0.08) |
| political interest | -0.01 | 0.11^***^ | 0.04 | 0.00 | 0.07^***^ | -0.00 |
|  | (0.01) | (0.02) | (0.02) | (0.01) | (0.01) | (0.02) |
| trust in government | 0.01 | 0.01 | 0.01 | -0.00 | -0.00 | 0.01 |
|  | (0.00) | (0.01) | (0.01) | (0.00) | (0.01) | (0.01) |
| government dissatisfaction | 0.01^*^ | 0.03^***^ | -0.00 | -0.00 | 0.01^*^ | 0.01 |
|  | (0.01) | (0.01) | (0.01) | (0.00) | (0.01) | (0.01) |
| indiv. economic threat | 0.02 | 0.08^*^ | 0.02 | 0.01 | 0.01 | 0.05^*^ |
|  | (0.02) | (0.04) | (0.04) | (0.01) | (0.02) | (0.03) |
| indiv. health threat | 0.03 | 0.02 | 0.05 | -0.00 | 0.00 | 0.01 |
|  | (0.02) | (0.04) | (0.04) | (0.01) | (0.03) | (0.03) |
| soc. economic threat | -0.09^***^ | -0.10^*^ | -0.14^**^ | -0.01 | -0.02 | -0.04 |
|  | (0.02) | (0.04) | (0.04) | (0.01) | (0.03) | (0.03) |
| soc. health threat | 0.01 | 0.00 | -0.04 | 0.01 | -0.01 | -0.01 |
|  | (0.02) | (0.04) | (0.04) | (0.01) | (0.03) | (0.03) |
| AIC | 239.80 | 1231.53 | 1314.51 | -1367.82 | 476.70 | 604.23 |
| BIC | 321.52 | 1313.24 | 1396.23 | -1286.10 | 558.42 | 685.95 |
| Log Likelihood | -102.90 | -598.76 | -640.25 | 700.91 | -221.35 | -285.12 |
| Deviance | 65.81 | 197.12 | 216.07 | 11.12 | 85.53 | 98.48 |
| Num. obs. | 904 | 904 | 904 | 904 | 904 | 904 |
| ^***^p < 0.001; ^**^p < 0.01; ^*^p < 0.05 | | | | | | |

Figure 6: Odds ratios of main variables for France


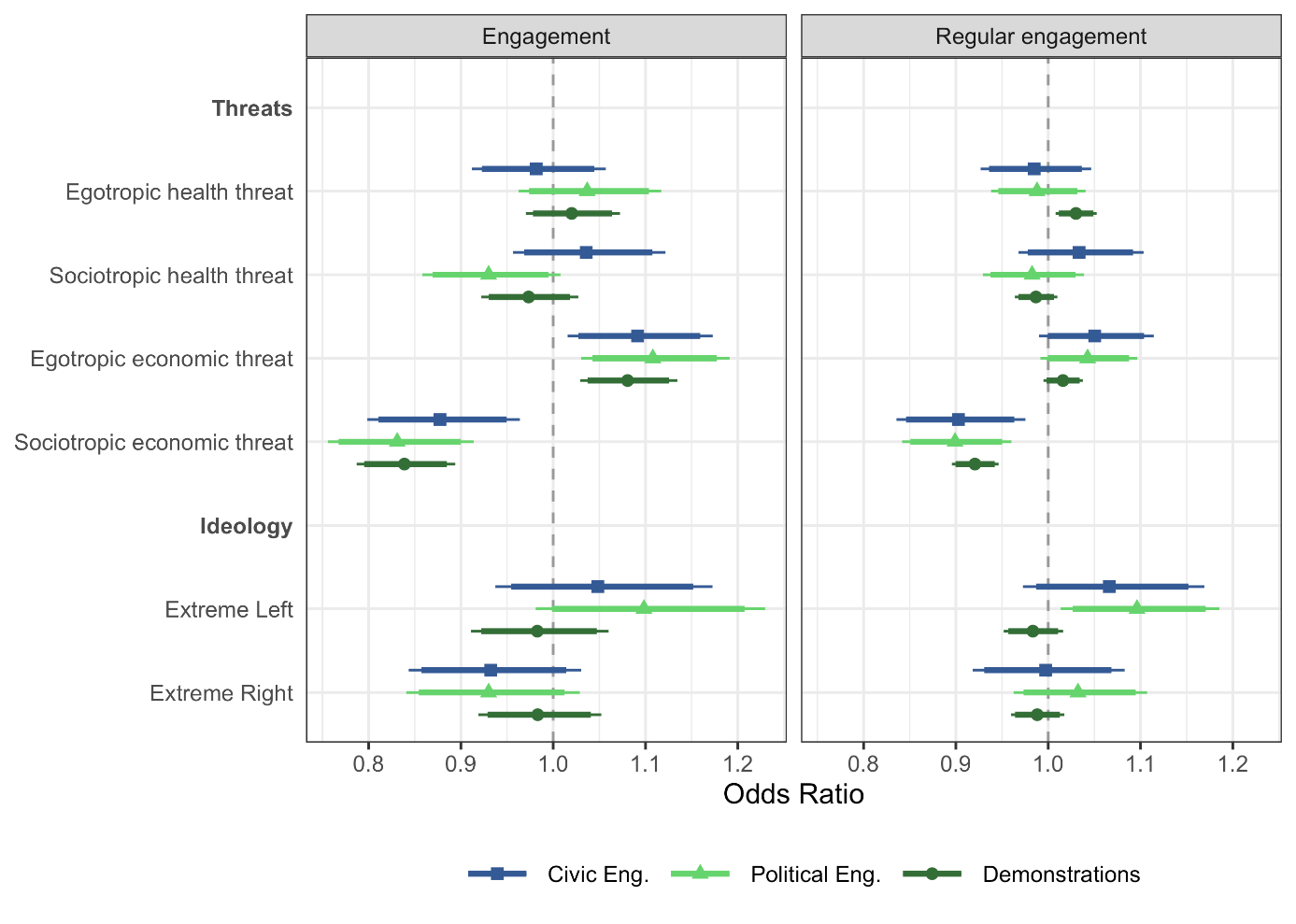


Table 8 models for France

|  | **Demonstration** | **Political** | **Civic** | **Demonstration - regular** | **Political -regular** | **Civic -regular** |
| --- | --- | --- | --- | --- | --- | --- |
| intercept | 0.24^*^ | 0.61^***^ | 0.36^*^ | 0.06 | 0.13 | -0.14 |
|  | (0.11) | (0.17) | (0.17) | (0.05) | (0.12) | (0.14) |
| age | -0.01 | -0.01^*^ | 0.00 | -0.00 | -0.01^*^ | 0.00 |
|  | (0.00) | (0.01) | (0.01) | (0.00) | (0.00) | (0.01) |
| age^2 | 0.00 | 0.00 | 0.00 | 0.00 | 0.00 | -0.00 |
|  | (0.00) | (0.00) | (0.00) | (0.00) | (0.00) | (0.00) |
| gender | -0.00 | 0.05 | 0.04 | 0.00 | 0.04 | 0.07^*^ |
|  | (0.02) | (0.03) | (0.03) | (0.01) | (0.02) | (0.03) |
| children | 0.03^**^ | 0.02 | 0.00 | 0.00 | 0.02 | -0.00 |
|  | (0.01) | (0.02) | (0.02) | (0.00) | (0.01) | (0.01) |
| education | -0.00 | 0.04 | 0.03 | -0.02 | 0.03 | 0.02 |
|  | (0.02) | (0.04) | (0.04) | (0.01) | (0.03) | (0.03) |
| economic situation | 0.00 | -0.00 | 0.02 | 0.01 | -0.01 | 0.02 |
|  | (0.01) | (0.02) | (0.02) | (0.01) | (0.01) | (0.02) |
| extreme left | -0.02 | 0.09 | 0.05 | -0.02 | 0.09^*^ | 0.06 |
|  | (0.04) | (0.06) | (0.06) | (0.02) | (0.04) | (0.05) |
| extreme right | -0.02 | -0.07 | -0.07 | -0.01 | 0.03 | -0.00 |
|  | (0.03) | (0.05) | (0.05) | (0.01) | (0.04) | (0.04) |
| political interest | 0.05^***^ | 0.14^***^ | 0.07^***^ | 0.02^**^ | 0.10^***^ | 0.03^*^ |
|  | (0.01) | (0.02) | (0.02) | (0.01) | (0.01) | (0.02) |
| trust in government | 0.01 | -0.01 | 0.01 | 0.00 | -0.01 | 0.01 |
|  | (0.01) | (0.01) | (0.01) | (0.00) | (0.01) | (0.01) |
| government dissatisfaction | 0.01 | 0.01 | -0.00 | -0.00 | 0.01 | 0.01 |
|  | (0.01) | (0.01) | (0.01) | (0.00) | (0.01) | (0.01) |
| indiv. economic threat | 0.08^**^ | 0.10^**^ | 0.09^*^ | 0.02 | 0.04 | 0.05 |
|  | (0.02) | (0.04) | (0.04) | (0.01) | (0.03) | (0.03) |
| indiv. health threat | 0.02 | 0.04 | -0.02 | 0.03^**^ | -0.01 | -0.02 |
|  | (0.03) | (0.04) | (0.04) | (0.01) | (0.03) | (0.03) |
| soc. economic threat | -0.18^***^ | -0.19^***^ | -0.13^**^ | -0.08^***^ | -0.11^**^ | -0.10^**^ |
|  | (0.03) | (0.05) | (0.05) | (0.01) | (0.03) | (0.04) |
| soc. health threat | -0.03 | -0.07 | 0.04 | -0.01 | -0.02 | 0.03 |
|  | (0.03) | (0.04) | (0.04) | (0.01) | (0.03) | (0.03) |
| AIC | 513.81 | 1239.81 | 1225.80 | -1014.58 | 575.04 | 868.91 |
| BIC | 595.66 | 1321.66 | 1307.65 | -932.73 | 656.89 | 950.75 |
| Log Likelihood | -239.91 | -602.91 | -595.90 | 524.29 | -270.52 | -417.45 |
| Deviance | 89.94 | 199.55 | 196.50 | 16.80 | 96.19 | 132.81 |
| Num. obs. | 911 | 911 | 911 | 911 | 911 | 911 |
| ^***^p < 0.001; ^**^p < 0.01; ^*^p < 0.05 | | | | | | |

Figure 7: Odds ratios of main variables for Spain


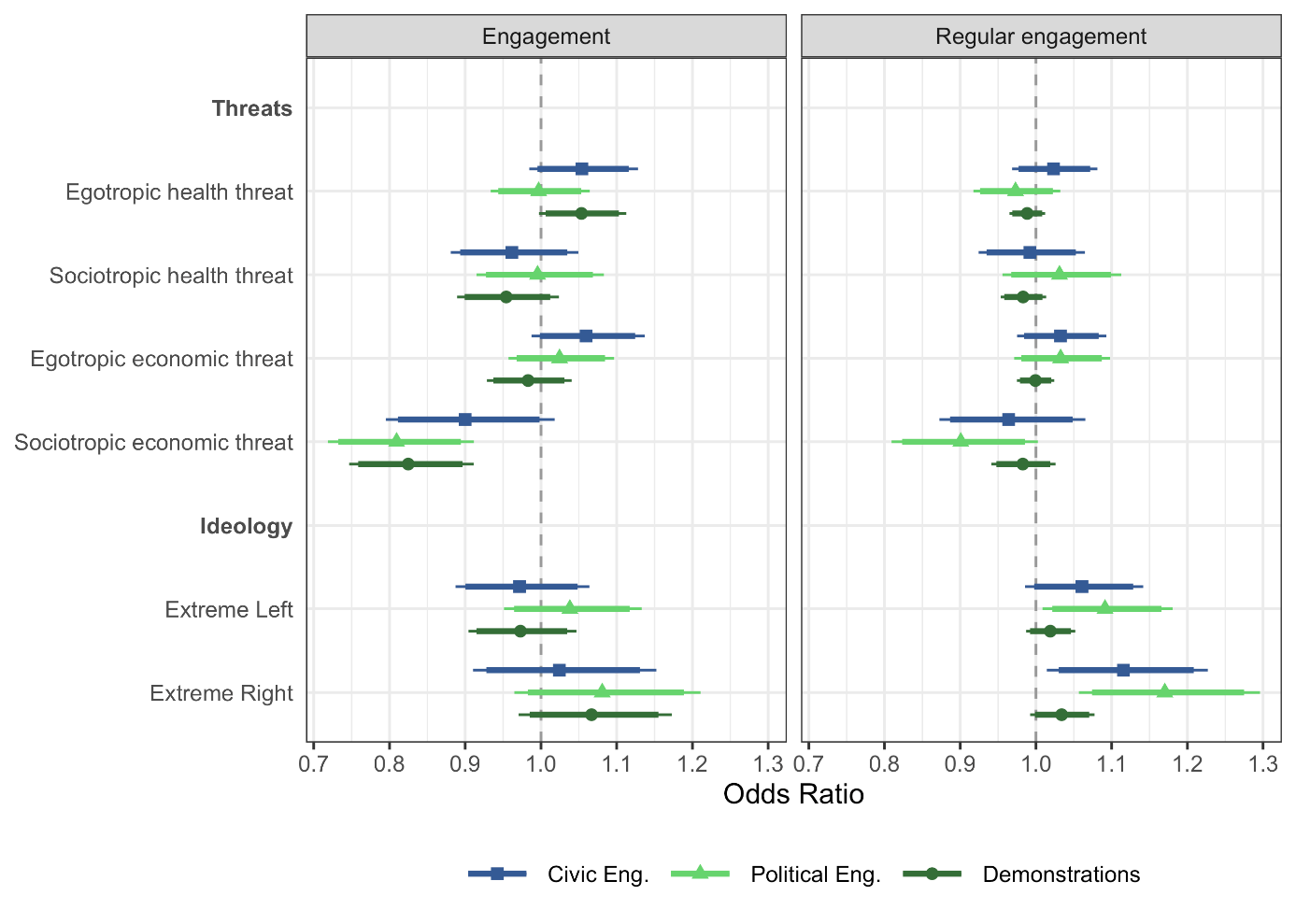


Table 9 models for Spain

|  | **Demonstration** | **Political** | **Civic** | **Demonstration - regular** | **Political -regular** | **Civic -regular** |
| --- | --- | --- | --- | --- | --- | --- |
| intercept | 0.27 | 0.46^**^ | 0.42^*^ | 0.07 | -0.04 | 0.07 |
|  | (0.15) | (0.18) | (0.18) | (0.06) | (0.16) | (0.15) |
| age | -0.00 | -0.01 | 0.00 | -0.00 | -0.01 | 0.00 |
|  | (0.01) | (0.01) | (0.01) | (0.00) | (0.01) | (0.01) |
| age^2 | 0.00 | 0.00 | -0.00 | 0.00 | 0.00 | -0.00 |
|  | (0.00) | (0.00) | (0.00) | (0.00) | (0.00) | (0.00) |
| gender | -0.01 | 0.06^*^ | 0.05 | -0.00 | 0.06^*^ | 0.05 |
|  | (0.03) | (0.03) | (0.03) | (0.01) | (0.03) | (0.03) |
| children | 0.06^***^ | 0.04^*^ | 0.03 | 0.01 | 0.03 | 0.04^**^ |
|  | (0.01) | (0.02) | (0.02) | (0.01) | (0.02) | (0.01) |
| education | 0.04 | 0.05 | 0.04 | 0.00 | 0.02 | 0.03 |
|  | (0.03) | (0.03) | (0.03) | (0.01) | (0.03) | (0.03) |
| economic situation | 0.01 | 0.02 | 0.02 | -0.00 | -0.00 | -0.01 |
|  | (0.02) | (0.02) | (0.02) | (0.01) | (0.02) | (0.02) |
| extreme left | -0.03 | 0.04 | -0.03 | 0.02 | 0.09^*^ | 0.06 |
|  | (0.04) | (0.04) | (0.05) | (0.02) | (0.04) | (0.04) |
| extreme right | 0.06 | 0.08 | 0.02 | 0.03 | 0.16^**^ | 0.11^*^ |
|  | (0.05) | (0.06) | (0.06) | (0.02) | (0.05) | (0.05) |
| political interest | 0.05^***^ | 0.14^***^ | 0.04^*^ | 0.02^*^ | 0.12^***^ | 0.01 |
|  | (0.01) | (0.02) | (0.02) | (0.01) | (0.02) | (0.01) |
| trust in government | 0.01 | 0.01 | 0.01 | 0.00 | 0.01 | -0.00 |
|  | (0.01) | (0.01) | (0.01) | (0.00) | (0.01) | (0.01) |
| government dissatisfaction | 0.00 | 0.00 | 0.00 | -0.00 | 0.01 | -0.01 |
|  | (0.01) | (0.01) | (0.01) | (0.00) | (0.01) | (0.01) |
| indiv. economic threat | -0.02 | 0.02 | 0.06 | -0.00 | 0.03 | 0.03 |
|  | (0.03) | (0.03) | (0.04) | (0.01) | (0.03) | (0.03) |
| indiv. health threat | 0.05 | -0.00 | 0.05 | -0.01 | -0.03 | 0.02 |
|  | (0.03) | (0.03) | (0.03) | (0.01) | (0.03) | (0.03) |
| soc. economic threat | -0.19^***^ | -0.21^***^ | -0.11 | -0.02 | -0.10 | -0.04 |
|  | (0.05) | (0.06) | (0.06) | (0.02) | (0.05) | (0.05) |
| soc. health threat | -0.05 | -0.00 | -0.04 | -0.02 | 0.03 | -0.01 |
|  | (0.04) | (0.04) | (0.04) | (0.02) | (0.04) | (0.04) |
| AIC | 848.36 | 1188.21 | 1259.74 | -727.70 | 988.51 | 856.13 |
| BIC | 930.81 | 1270.66 | 1342.20 | -645.25 | 1070.96 | 938.58 |
| Log Likelihood | -407.18 | -577.11 | -612.87 | 380.85 | -477.26 | -411.06 |
| Deviance | 130.27 | 186.72 | 201.42 | 24.53 | 151.12 | 131.35 |
| Num. obs. | 944 | 944 | 944 | 944 | 944 | 944 |
| ^***^p < 0.001; ^**^p < 0.01; ^*^p < 0.05 | | | | | | |

# A5: Additional figures and tables

Figure 1: Level of engagement across countries


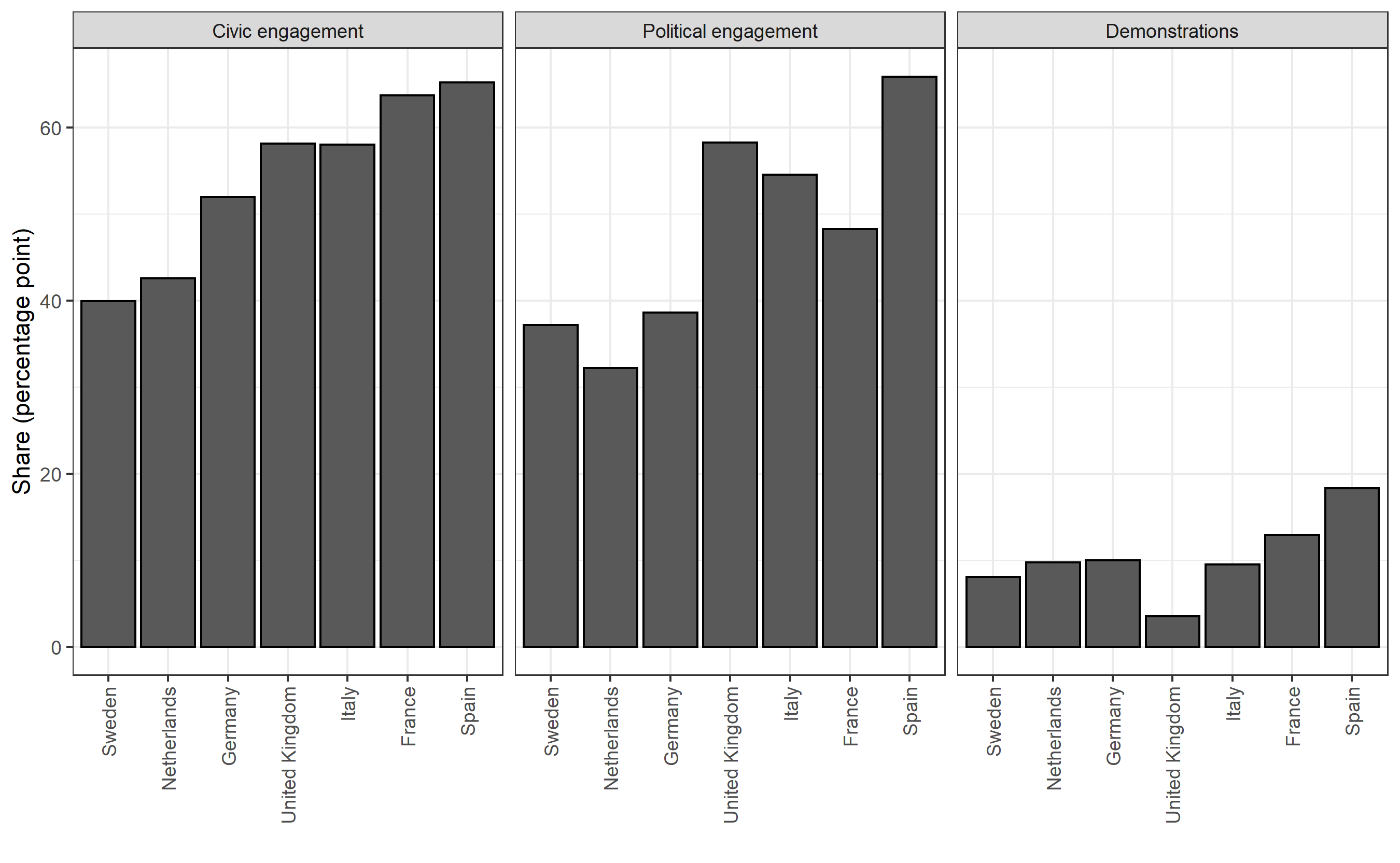


Table 1: Level of engagement by form and country

| **Country** | **Indicator** | **Overall engagement share** | **Regular engagement share** |
| --- | --- | --- | --- |
| France | Contacted a pol. | 14.7 | 2.4 |
|  | Donated money | 28.9 | 4.7 |
|  | Help in neighborhood | 59.3 | 15.5 |
|  | Other political | 18.6 | 3.4 |
|  | Posted on the Internet | 31.5 | 8 |
|  | Public demonstration | 13 | 1.9 |
|  | Signed a petition | 32.8 | 7.1 |
| Germany | Contacted a pol. | 12.5 | 2.4 |
|  | Donated money | 27 | 4.3 |
|  | Help in neighborhood | 44.9 | 10.2 |
|  | Other political | 16 | 3.3 |
|  | Posted on the Internet | 24.4 | 6.6 |
|  | Public demonstration | 10 | 1.8 |
|  | Signed a petition | 25.5 | 5 |
| Italy | Contacted a pol. | 15.5 | 2 |
|  | Donated money | 45.3 | 6 |
|  | Help in neighborhood | 37.6 | 7.2 |
|  | Other political | 13.5 | 2.7 |
|  | Posted on the Internet | 42.6 | 11.5 |
|  | Public demonstration | 9.5 | 1.4 |
|  | Signed a petition | 29.1 | 5 |
| Netherlands | Contacted a pol. | 10 | 1.9 |
|  | Donated money | 28.6 | 4.7 |
|  | Help in neighborhood | 34.4 | 6.9 |
|  | Other political | 17.2 | 3.3 |
|  | Posted on the Internet | 20.2 | 3.3 |
|  | Public demonstration | 9.8 | 1.5 |
|  | Signed a petition | 22.8 | 3.8 |
| Spain | Contacted a pol. | 15.4 | 2.2 |
|  | Donated money | 43.8 | 7.9 |
|  | Help in neighborhood | 56 | 13 |
|  | Other political | 18.3 | 4.2 |
|  | Posted on the Internet | 48.7 | 16 |
|  | Public demonstration | 18.3 | 2.5 |
|  | Signed a petition | 43.9 | 10.8 |
| Sweden | Contacted a pol. | 11.9 | 1.7 |
|  | Donated money | 23.2 | 2.8 |
|  | Help in neighborhood | 31.9 | 6.6 |
|  | Other political | 15.1 | 4.1 |
|  | Posted on the Internet | 29.2 | 9.1 |
|  | Public demonstration | 8.1 | 1.2 |
|  | Signed a petition | 20.2 | 3.9 |
| United Kingdom | Contacted a pol. | 15.5 | 2.3 |
|  | Donated money | 41.4 | 7.7 |
|  | Help in neighborhood | 39.2 | 8.2 |
|  | Other political | 13.5 | 2.9 |
|  | Posted on the Internet | 41.6 | 12.6 |
|  | Public demonstration | 3.6 | 0.6 |
|  | Signed a petition | 41.3 | 8.8 |

Table 2: Sample Size

| **Country** | **Sample size** |
| --- | --- |
| France | 1033 |
| Germany | 1036 |
| Italy | 1067 |
| Netherlands | 1103 |
| Spain | 1061 |
| Sweden | 1110 |
| United Kingdom | 1169 |
